# A Qualitative Comparative Analysis of the Drivers of HIV Status Knowledge in Orphans and Vulnerable Children in Mozambique

**DOI:** 10.9745/GHSP-D-20-00311

**Published:** 2020-09-30

**Authors:** Allie Davis, Zola Allen, Nena do Nascimento, Jenifer Chapman, Rotafina Donco, Daan Velthausz

**Affiliations:** a American Institute of Physics State Department Science Fellow, US Department of State, Washington, DC, USA.; b Palladium, Chapel Hill, NC, USA.; c Palladium, Washington, DC, USA.; d Maraxis, Maputo, Mozambique.

## Abstract

In Mozambique, more than a million children are living with HIV or are otherwise vulnerable due to HIV. In response to this crisis, the US President’s Emergency Plan for AIDS Relief funds programs that serve orphans and vulnerable children affected by HIV and their families. These programs retain case workers, known as activistas, who provide and refer beneficiaries to services to increase beneficiaries’ knowledge of their HIV status and to improve retention in care among those living with HIV. To improve program effectiveness, implementing organizations need to understand how different case management attributes affect beneficiary outcomes. We applied fuzzy-set qualitative comparative analysis (QCA), based on 119 interviews, to identify the combinations of case management attributes that led to (1) increased knowledge of HIV status, and (2) high percentages of beneficiaries with known HIV status. We identified 6 pathways for the first outcome and 5 pathways for the second outcome. Each pathway demonstrates an alternative combination of conditions that positively influences the outcome and is equally sufficient in achieving the outcome. To improve knowledge of HIV status, programs in a similar context as this study may select any of the identified pathways based on their existing resources and work on ensuring the presence of each of the conditions in the pathway. Overall, based on the presence of some of the factors in multiple pathways, we conclude that to improve knowledge of HIV status it is important that programs implement a formal process to assign cases considering case complexity and existing caseload to reduce overwork; provide activistas with external support such as weekly care team meetings, weekly supervisor meetings, and/or low supervision ratios; hire experienced activistas; provide all activistas with follow-up trainings so they have the tools to address challenging cases; expand the financial resources offered to activistas; and reimburse activistas for work-related expenses.

## Abstract (precis)

We identified combinations of modifiable factors that HIV programs supporting orphans, vulnerable children, and their families may be able to act on to increase the proportion of beneficiaries who know their HIV status.

## Teaser

**Key Findings:**

- Using fuzzy-set qualitative comparative analysis, we identified combinations of modifiable factors that HIV programs that support orphans, vulnerable children, and their families may be able to act on to increase the proportion of beneficiaries who know their HIV status.

**Key Implications:**

To improve HIV status knowledge, programs should consider implementing a formal process to:

- Assign cases considering case complexity and caseload to reduce overwork
- Provide activistas with supervision support
- Hire experienced activistas
- Train activistas to address challenging cases
- Reimburse activistas for work-related expenses

Resumo em português no final do artigo.

## INTRODUCTION

The HIV epidemic has exacted a formidable toll on children and their families. Currently, 13.4 million children are living without 1 or both parents due to the HIV epidemic; 80% of these children live in sub-Saharan Africa.^1^ In addition, 1.8 million children under age 15 are living with HIV.^2^ Despite some decline in HIV adult prevalence worldwide and increasing access to treatment, the number of children affected by or vulnerable to HIV remains alarmingly high. In response to the global HIV/AIDS epidemic, the United States initiated the President’s Emergency Plan for AIDS Relief (PEPFAR) in 2003. PEPFAR funds health care facilities, nongovernmental organizations, and other programs that provide support and services to populations vulnerable to or infected with HIV.^3^ In many countries with a high burden of HIV, PEPFAR supports programming for treatment and support to orphans and vulnerable children (OVC) and their families, especially those made vulnerable by HIV.^3^^,^^4^

The primary mechanism for service delivery and support for OVC programming is client case management. Individual orphans, vulnerable children, or their family members are enrolled into a program, and a case worker is assigned. The case worker assesses client needs, outlines a care plan and actions to achieve that care plan, monitors care plan achievement, and ultimately exits the client from the program upon care completion. A critically important aspect of case management is that case managers advocate on behalf of their clients, ensuring that medication is received, patient calls are returned, paperwork is filed with the clinic or care agency, and HIV- and non-HIV-related needs are met.^5^ Case workers are volunteers or stipend-paid community members who are trained by the program to provide services to clients, but they otherwise have little or no formal social work training or qualifications. Case workers are employed by a community-based organization (CBO) or implementing partner. CBOs often employ case worker supervisors whose role is to review client files with case workers, support them in meeting clients’ needs, support time management, assess training needs, identify training opportunities, and provide support to help them cope with job stress.

Client case management has been shown to positively influence key HIV outcomes, notably knowledge of HIV status and antiretroviral therapy (ART) retention.^6^^–^^8^ Studies have shown that increased contact with a case manager is related to a decreased need for income assistance, emotional counseling, and other forms of supportive services and increased access to health care.^7^^,^^9^^–^^11^ HIV programs are increasingly turning to family-oriented, case management approaches because of their potential to provide holistic, individualized, and effective support, particularly among vulnerable populations.^5^^,^^11^

Although case management is widely accepted as an effective method for HIV service provision, little is known about the specific attributes that make a case management program effective.^12^ Most studies that have evaluated case management programs have focused solely on how well these programs achieved final outcomes, such as knowledge of HIV status, ART retention, or cost effectiveness; they did not investigate the factors that led to those outcomes.^6^^,^^7^ The limited number of studies that have examined specific case management attributes primarily consider a small number of factors in isolation and do not account for how factors may combine to have a collective impact on program outcomes. From previous studies, several case management program attributes have been hypothesized to positively influence case management effectiveness, such as case manager skills,^13^^,^^14^ training,^9^^,^^15^^–^^17^ caseload,^18^ supportive supervision,^14^^,^^16^ financial incentives,^19^^–^^21^ resources,^19^^–^^21^ networking,^5^^,^^6^^,^^14^^,^^15^ and accessibility of care.^9^^,^^18^^,^^21^ Although there are many factors posited to influence knowledge of HIV status, such as stigma,^19^^,^^20^ demographics (e.g., beneficiary age, sex, income, education),^18^^,^^22^^,^^23^ and social capital,^15^ this study focused solely on the modifiable attributes of case management programs because these factors are in programs’ manageable control. The limited research on case management attributes that improve HIV outcomes highlights the need for research that comprehensively and specifically evaluates which aspects of case management programs influence case management effectiveness.

We used qualitative comparative analysis (QCA) to identify the modifiable case management attributes that optimize program performance within a PEPFAR-funded program in Mozambique. We defined optimized program performance as a scenario in which the proportion of beneficiaries that know their HIV status is increasing, which is a commonly used indicator in HIV programming.^7^ Specifically, this study identified the combinations of modifiable case management attributes that influenced 2 indicators (i.e., outcomes) of effectiveness: (1) percentage change in knowledge of HIV status, and (2) percentage of beneficiaries with HIV status known at the time of the last assessment. The results of this study contribute to a theory of effective OVC programs, identifying actionable recommendations that implementing organizations can follow to optimize program performance.

We used qualitative comparative analysis to identify the modifiable case management attributes that influenced percentage change in knowledge of HIV status and percentage of people with HIV status known.

## METHODS

### Research Context and Case Selection

In Mozambique, approximately 15% of women and 10% of men ages 15–49 are living with HIV.^24^ Under the age of 15, approximately 200,000 children in Mozambique are living with HIV, and 916,000 are considered vulnerable because of HIV prevalence.^25^ Low rates of treatment retention, especially among children, adolescents, and young adults, threaten to undermine epidemic control.^4^ Due to the high prevalence and risk for HIV in Mozambique, the United States Agency for Inter-national Development (USAID) funds OVC-focused programming through a program called COVida.^26^ The program partners with CBOs to recruit and equip case managers to provide services to OVC and their families and is a major center of case management in Mozambique.^26^ COVida-affiliated CBOs are the focus of this study. The unit of analysis for this study is the case manager, called activista in Mozambique (and referred to as such herein). Activistas are expected to work about 20 hours weekly. Across all COVida CBOs, activistas have managed 344,000 beneficiaries, 60% of whom are currently active.^27^

For QCA, it is important that the units of analysis exhibit varied degrees of the outcome(s) and the factors analyzed. COVida has CBOs in all 11 provinces in Mozambique. To gather in-depth knowledge about each program, we selected 3 provinces: Maputo, Gaza, and Nampula. The 3 provinces were selected based on the following considerations: percentage of children living with HIV, percentage of children on ART, number of COVida beneficiaries, number of COVida beneficiaries who were living with HIV, USAID priority status for a province, program stability, and security. Within each province, we selected 2 CBOs: 1 CBO with a high proportion of beneficiaries with unknown HIV status, and 1 CBO with a low proportion of beneficiaries with unknown HIV status. In each selected CBO, we randomly sampled 11 or 12 activistas and then interviewed their managers (activista chefes) and supervisors.

### Data Collection Methods

Data were collected using qualitative and quantitative methods. From each CBO, 20 people were interviewed: 11 or 12 activistas, 3 activista chefes (i.e., direct manager of activistas), 2 supervisors (i.e., manager of activista chefes), and 3 other management staff. To be included, an activista must have been working for COVida for at least 6 months, and their activista chefe and supervisor had to be available for interviews. In total, 70 activistas, 18 activista chefes, 12 supervisors, 6 CBO managers, 6 CBO monitoring and evaluation advisors, and the COVida project director were interviewed. Interviews were 30–45 minutes long, and all interviews were audio recorded and conducted privately at the CBOs. Data collection was done by a local research agency. Data collectors were selected based on level of education, prior qualitative interview experience, knowledge of the study areas, and fluency in study languages. A gender balance was ensured during recruitment of data collectors. Data collectors received 4 days of study-specific data collection protocol training. The data collection protocol was pilot tested and revised before conducting fieldwork. Data collection took place from July to August 2019.

In total, 70 activistas, 18 activista chefes, 12 supervisors, 6 CBO managers, 6 CBO monitoring and evaluation advisors, and the COVida project director were interviewed.

Interviews elicited information including activista caseload, training, supervision, team meetings, nonmonetary incentives, networking, demographics, work satisfaction, ways to improve service quality, time spent working, and any costs they incurred. Activista chefe and supervisor interviews also discussed challenges with activista retention, salaries, and activista performance. In interviews with CBO managers, advisors, and the project director, res-pondents discussed CBO-level procedures. Docu-mentation was collected such as project reports, quarterly updates, and routine project data collected by COVida. Routine data tracked by COVida included activista caseload and beneficiary HIV status.

The study protocol received institutional review board approval from the Comitê Nacional de Bioética para a Sáude in Mozambique and Health Media Labs, Inc. in Washington, DC. The informed consent process for interview participants was individualized and private. The data collectors privately shared information about the study with each potential participant and obtained and documented a written informed consent. Informed consent was administered in the language preferred by the participant. If consent was granted for audio recording, we recorded the interview. The information provided by respondents was held in strict confidence.

### Data Analysis

#### Preliminary Analyses

All interviews were translated and transcribed. The transcripts were qualitatively coded, a process whereby common and relevant themes are identified and sections of the transcripts that relate to the themes are tagged.^28^ The qualitative coding employed a deductive approach, in which topics related to modifiable case management attributes were identified by program experts and stakeholders before coding began.^29^ For example, “training” was identified as a potentially important theme, because comprehensive training has been shown to positively influence program outcomes in health^15^ and resource-limited contexts.^30^^,^^31^ Microsoft Excel was used for the coding. Next, the coded data were reviewed, and summaries of each theme were created for each activista, activista chefe, and supervisor. The summaries were compared across each of these 3 roles to identify and resolve conflicts. Conflicting statements were resolved by triangulating data from interviews and documentation, to ensure internal validity.^32^ The summaries were also used to identify differences between activistas across the modifiable attributes. This preliminary qualitative analysis was essential to set up the QCA.^32^

Additionally, quantitative data from the interviews were analyzed. Descriptive statistics were calculated, using Microsoft Excel and SAS version 9.4, to summarize the range, mean, median, mode, frequency, and cumulative average of quantitative variables. These statistics were important to understand the spread of data across activistas for each modifiable attribute and to identify differences among activistas.

#### Qualitative Comparative Analysis

To identify the combinations of modifiable attributes that influenced knowledge of HIV status, a QCA was conducted. QCA combines quantitative and qualitative analyses to determine which combinations of variables (called pathways) influence the outcome analyzed.^33^^–^^35^ We selected QCA for this study since this method recognizes that several different combinations of variables may lead to a particular outcome. As a result, an implementing partner may choose any of the identified pathways to improve outcomes of interest. In QCA, the variables are referred to as causal conditions and are similar to independent variables in a statistical analysis.^35^ For this study, the modifiable attributes of case management are the causal conditions (e.g., caseload). The outcomes are similar to dependent variables and are the phenomena that are the main focus of the study.^35^ Because we did not want to lose information from data by restricting all conditions to dichotomous values, fuzzy-set QCA (fsQCA) was the analysis method selected, whereby fuzzy sets that ranged continuously from 0 to 1 were used to measure varying degrees of a condition’s presence or absence.^34^

#### Outcome Identification and Calibration

Two outcomes were investigated for this study: (1) percentage change in knowledge of HIV status, and (2) percentage of beneficiaries with HIV status known at the time of the last assessment. Also, we conducted the analysis of the negation of the outcomes. We investigated the conditions that do not produce a high percentage change in knowledge of HIV status and conditions that produce high percentage of beneficiaries with HIV status unknown at the last assessment. The outcomes were calibrated using the direct calibration approach (see the QCA calibration guide in Supplement), in which a quantitative value associated with in-set membership, out-of-set membership, and the crossover point is first identified based on theory and the distribution of the raw, quantitative data; the data are then normalized between these points.^36^ Calibration is an iterative process between theory and collected data that aims to develop a common measuring stick to use to determine whether a case falls in the set of a phenomenon, out of the set of a phenomenon, or somewhere in between.^32^ The first outcome investigated was the percentage of an activista’s beneficiaries who changed their reported HIV status from unknown to known between enrollment and July 2019. These beneficiaries included all those who enrolled before April 1, 2019, and had their HIV status recorded at least 1 time in addition to the time of enrollment. HIV status was considered known if the beneficiary’s status was documented as HIV positive, on ART, not on ART (likely HIV positive but not receiving treatment), or test not recommended (likely not HIV positive, based on a risk assessment). HIV status was considered unknown if the beneficiary’s status was documented as unknown or not revealed. In-set membership was when the percentage of an activista’s beneficiaries with a change in HIV known status was greater than or equal to 75%, and out-of-set membership was when the percentage with a change in HIV known status was less than or equal to 25%.

The second outcome investigated was the percentage of an activista’s beneficiaries with their HIV status known at the last assessment. HIV status was considered known for beneficiaries whose status was either HIV positive or negative (and was not documented as unknown or not revealed) at the time of the last assessment. Beneficiaries for both second and third outcomes included all those who enrolled before April 1, 2019. In-set membership was when the percentage of an activista’s beneficiaries with HIV status known was greater than or equal to 95%, and out-of-set membership was when the percentage of an activista’s beneficiaries with HIV status known was less than or equal to 75%.

#### Identification and Preliminary Removal of Potential Causal Conditions

A list of 23 potential causal conditions that were modifiable case management attributes was assembled from literature, case knowledge, and project documentation. Twenty-three causal conditions are too many for 70 cases in QCA because too much of the logic space or all of the possible combinations of conditions would not be represented by empirical cases.^34^ Conditions were removed based on lack of variation across the cases within a condition (known as domain conditions)^34^^,^^35^; correlations with other conditions, indicating that 2 conditions may be measuring the same topic^31^^,^^34^; lack of data^32^^,^^34^; or low necessity, indicating that the condition was less important for the outcome).^34^^,^^37^ Necessity is a QCA metric used to analyze individual conditions and can be helpful to narrow down a large list of potential conditions.^35^ Necessity reflects how important a condition is for an outcome, based on how often the condition is present when the outcome is present^34^; necessity scores between 0.9 and 1.0 indicate that a condition is almost always necessary for the outcome.^34^

**FIGURE 1.**
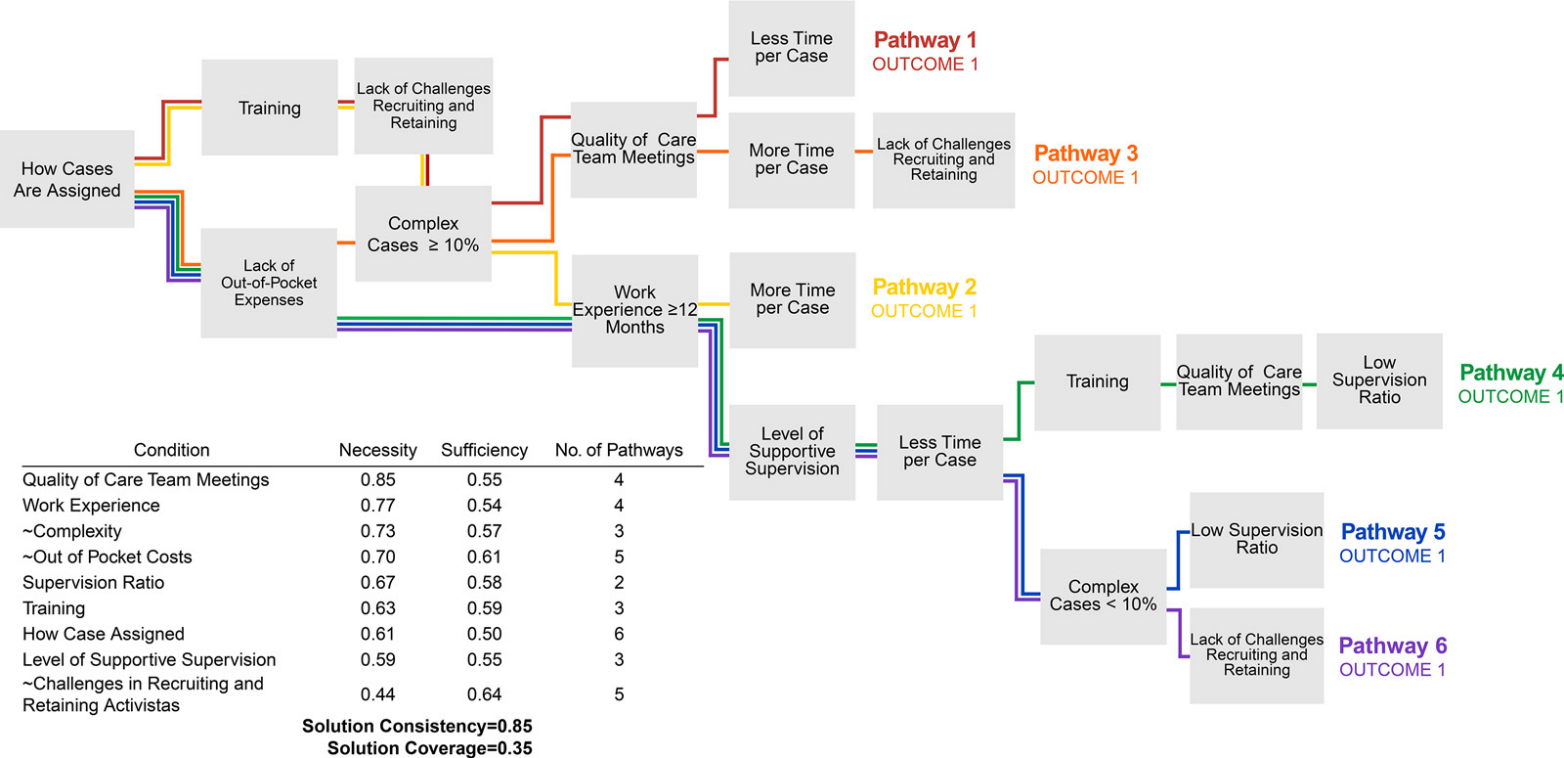
Outcome 1: Percentage Change in HIV Status Knowledge in Orphans or Vulnerable Children in Mozambique Note: The numbering of the pathways is random; each pathway demonstrates an alternative combination of conditions that positively influenced the outcome and is considered to be equally sufficient in achieving the outcome.

**FIGURE 2.**
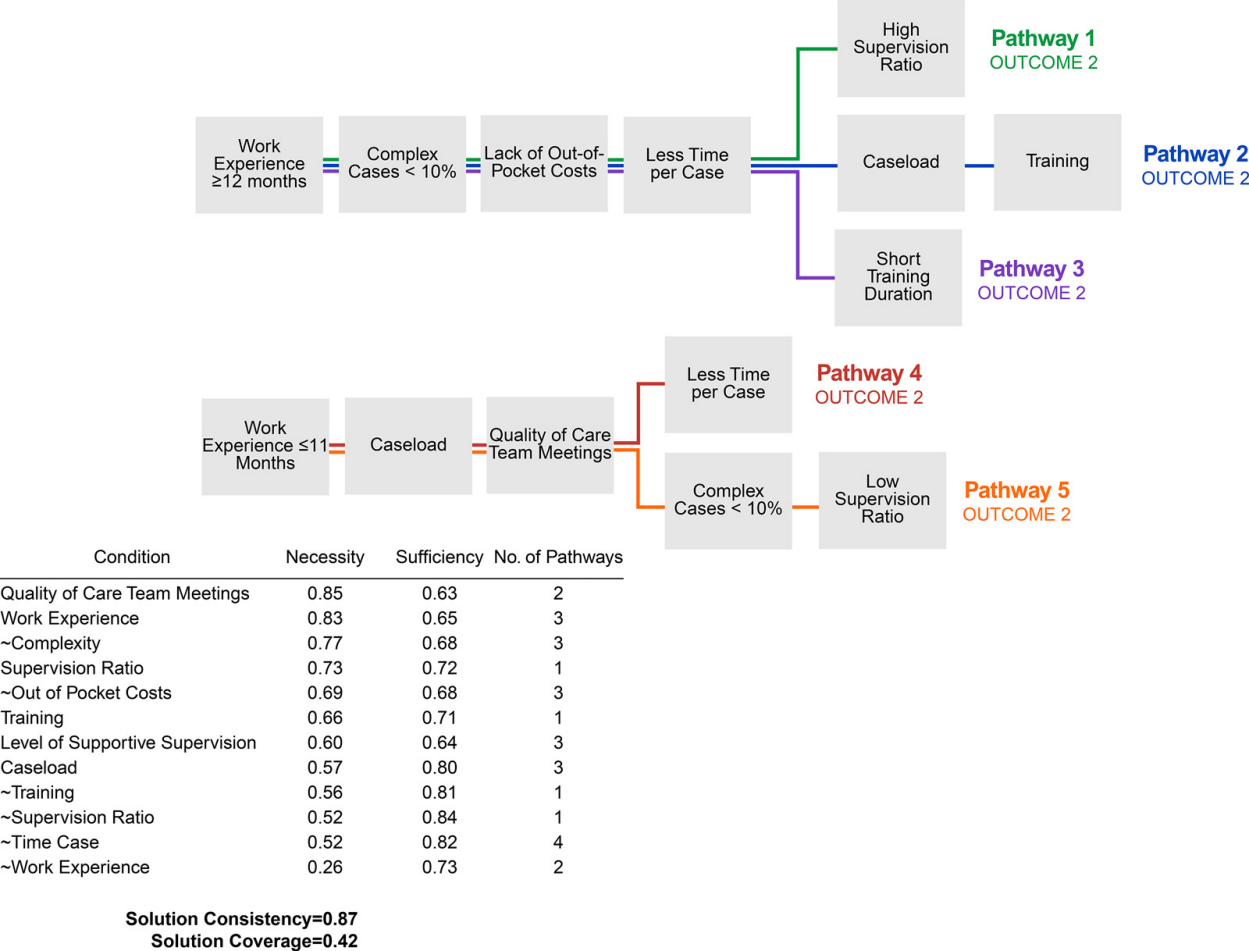
Outcome 2: Percentage of Orphans or Vulnerable Children in Mozambique With HIV Status Known

**FIGURE 3.**
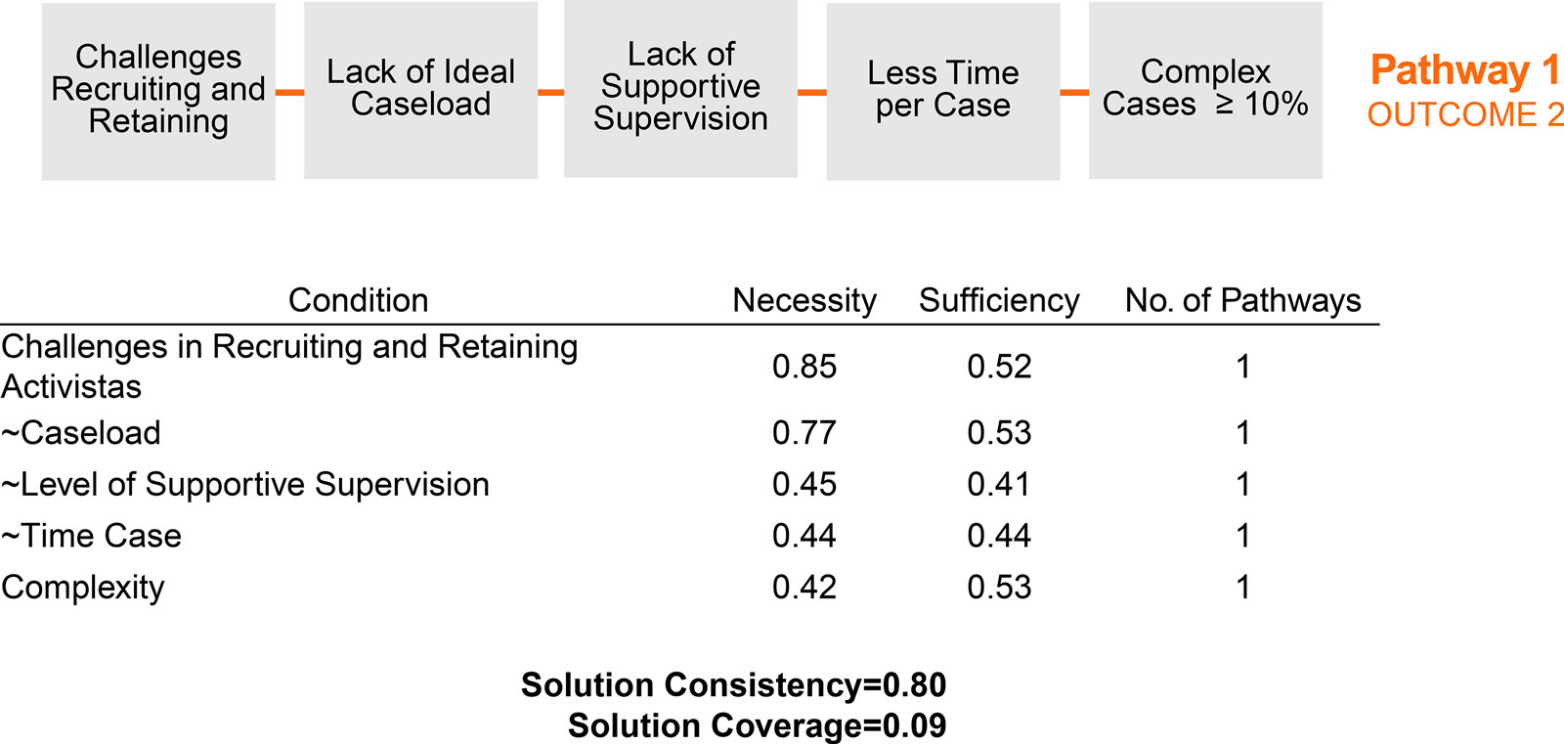
Outcome 2 Negation Analysis: Percentage of Orphans or Vulnerable Children in Mozambique With HIV Status Unknown

Sufficiency reflects the extent to which a condition contributes to the presence of the outcome^36^; a sufficiency score above 0.8 is required for a condition to be sufficient alone to produce the outcome.^34^ Necessity and sufficiency scores are produced by the software at the analysis stage. We provide both necessity and sufficiency scores in Figures 1–3. As a check on the completeness of each final solution, the removed conditions were added back in and never resulted in higher solution consistency or coverage. From these initial analysis steps, the number of causal conditions analyzed was reduced to 11, which is a reasonable number for 70 cases using QCA^34^: caseload, complexity, challenges in recruiting and retaining activistas, how cases are assigned, level of supportive supervision, out-of-pocket costs, quality of care team meetings, supervision ratio, time spent per case, training, and work experience.

#### Calibration of Causal Conditions

Two methods were used to calibrate the causal conditions. First, the indirect calibration method was used for conditions that primarily had qualitative data^32^: challenges recruiting and retaining, how cases are assigned, level of supportive supervision, out-of-pocket costs, and training. Time spent per case was also calibrated indirectly, using primarily quantitative data.^34^^,^^36^ Project reports, documentation, literature, and expert knowledge were used to establish initial definitions for in-set membership (the criteria that correspond with a value of 1, when the condition is fully present for a given case or the case has full membership in the set of that condition), out-of-set membership (the criteria that correspond with a value of 0, when the condition is fully absent for a given case or the case has full nonmembership in the set of that condition), and the crossover point (the criteria that correspond with a value of 0.5, when the condition is neither present nor absent and is the point of maximum ambiguity). Next, qualitative data summaries were reviewed to determine meaningful differences between the activistas. For most of the indirectly calibrated conditions, 4-value fuzzy sets were used: 0 (fully out of the set), 0.33 (more out of the set than in), 0.67 (more in the set than out), and 1 (fully in the set); these sets are very common for fsQCA.^32^^,^^34^ For example, for the condition of challenges recruiting and retaining, in-set membership (a fuzzy set value of 1) was defined as:

*The care team reports significant issues with recruiting and retaining activistas, is understaffed, and lacks a clear plan to recruit and retain activistas.*

A value of 0.67 was defined as:

*There are many issues with activista recruiting or retention, and activistas leave for reasons beyond the low subsidy. The care team may have plans to alleviate activista turnover, but no action has been taken.*

A value of 0.33 was defined as:

*There are some issues with activista recruiting or retention, such as activistas leaving due to low subsidies. The care team demonstrates clear actions and plans devised to alleviate activista turnover.*

Out-of-set membership (a value of 0) was defined as:

*The entire care team does not report issues with recruiting or retaining activistas, is fully staffed, and has a clear plan in place to recruit and retain activistas.*

The remaining conditions, caseload and complexity, were calibrated using the direct calibration method, which is common for conditions with only quantitative data that can be normalized between anchor points.^36^ We measured complexity as the proportion of beneficiaries living with HIV in the activista’s case load. It was important to include complexity in our analysis since our interviews demonstrated that beneficiaries living with HIV often require more time from social workers to address issues such as comorbidities or treatment adherence, which would leave less time for other beneficiaries.

#### Truth Table Assembly

Once the causal conditions and outcomes were calibrated, the calibration criteria were used to assign fuzzy values to each case, for every condition and outcome. The qualitative coding and summaries and the quantitative values were used to determine whether each activista met the required criteria for in-set membership, out-of-set membership, or a membership value in between. These fuzzy values were assembled in a truth table that summarized the fuzzy scores assigned to causal conditions and outcomes for all activistas, reflecting the possible configurations of causal conditions associated with outcomes.^34^

#### Truth Table Analysis

Following the calibration of the causal conditions and outcomes, the truth table was analyzed using the “truth table analysis” function in fs/QCA software.^38^ Truth table analysis relies on the process of minimization, whereby stepwise comparisons between each combination of conditions are performed to determine which conditions could be removed to provide a more simplified pathway that consistently leads to the outcome.^34^^,^^38^ All possible combinations of conditions were investigated to determine (1) whether a given group of conditions consistently (i.e., nearly always) led to (i.e., was present when) the outcome occurred; and (2) whether the consistent combinations made sense with in-depth knowledge of the data (e.g., it would **not** make sense for challenges recruiting and retaining activistas to be a factor that contributed to a high percentage change in beneficiary HIV known status, but it **would** make sense for high-quality care team meetings to positively affect this outcome). Additionally, to further simplify the pathways, “easy” counterfactuals were used, called simplifying assumptions in QCA^34^; this action allows the researcher to specify assumptions for whether a causal condition’s presence or absence would be expected to be associated with each outcome. We made these as-sumptions based on case knowledge and theory of whether the presence or absence of a condition will lead to the outcome of interest. Thus, we assumed that presence of training will lead to high proportion of beneficiaries who know their HIV status. When there was not sufficient evidence to suggest the directionality of the condition’s influence on the outcome, both the presence and the absence of the condition were analyzed. For example, more time spent with each household could lead to a better outcome or it could lead to activista burnout and subsequently a poor outcome.

The validity of the results was determined based on 2 important QCA metrics: consistency and coverage. Consistency demonstrates the relative frequency that a pathway will result in a particular outcome or how **consistently** a pathway leads to that particular outcome; the accepted cutoff value for a consistent pathway is 0.8.^39^ Cover-age is the percentage of cases with an outcome that is explained by a given pathway.^40^ Coverage lacks a cutoff value because it is a metric for generalizability^39^; lower coverage scores, however, reflect more case-specific and less generalizable results.^34^ Once preliminary results were obtained, a subset/superset analysis was performed for each outcome to further simplify the number of causal conditions in each pathway while maintaining or increasing each pathway’s consistency and coverage.^34^ Finally, pathways were compared with theory and case knowledge to ensure that the final solutions presented the most complete and simplified explanations for the outcomes analyzed.

## RESULTS

### Study Participants

We interviewed 70 activistas, 18 activista chefes, 12 supervisors, and 6 CBO managers. The majority of activistas (71.4%) were women. The mean age was 30 years (standard deviation [SD]=9, range=20–57 years). Half of the activistas served beneficiaries in rural areas (51.4%). The majority of activistas (72.9%) had secondary education, one-quarter (24.3%) had primary education, and 3% had technical or professional education. Activistas’ total time working ranged from 6 months to 8 years, and 82.86% had 2 years of experience or less.

More than half of the activista chefes (55.6%, N=18) were women. The mean age was 34 years (SD=8.3, range=21–51 years). The majority of activista chefes (77.8%, N=18) had secondary education, less than a fifth (16.7%) had primary education only; 6% had technical or professional education. The number of years of work as activista chefes ranged from 6 months to 3 years. The majority of supervisors (91.7%, N=12) were men. The mean age was 40 years (SD=10.5, range=28–58 years). Two-thirds of supervisors (66.7%, N=12) had secondary education, and one-third (33.3%) had a university degree. The number of years of work as supervisors ranged from 6 months to 2 years. Lastly, 4 of the 6 CBO managers were women. The mean age was 51 years (SD=16.5, range=27–66 years). All 6 respondents had worked as CBO managers for 2 years.

### Outcome 1: Percentage Change in HIV Status Knowledge

For Outcome 1, 6 pathways led to a high percentage change in beneficiaries’ HIV status knowledge (Figure 1). Each pathway is 1 branch, with 5–8 conditions. For example, from Figure 1, Pathway 1 contained the following conditions: how cases are assigned, training, lack of challenges recruiting and retaining, complexity, quality of care team meetings, and less time per case. The solution consistency was 0.85 and coverage was 0.35. These pathways described the conditions that led to improved knowledge of HIV status for activistas from CBOs 2, 3, 4, and 6. Although some of the pathways are complicated (i.e., with more than 5 conditions), the solution reflects the most simplified combinations of conditions that consistently led to a high percentage change in HIV known status. No single condition was necessary or sufficient; instead, the combinations of conditions had the most important influence on the outcome.

No single condition was necessary or sufficient for Outcome 1; instead, the combinations of conditions had the most important influence on the outcome.

#### Common Conditions

One condition was shared by all 6 pathways: how cases are assigned. This finding demonstrates that decisions to assign new cases should consider activista caseload, work experience, skills, case complexity, and activista proximity to case to prepare activistas well for effective case management. In particular, consideration of caseload and complexity of cases ensured that activistas were not overburdened and had enough time to address the needs of each beneficiary. In contrast, activistas who did not achieve high percentage changes in HIV known status were part of programs that lacked a formal procedure to assign cases. For example, an activista chefe from CBO 5 stated:

*The communities are divided into boroughs and the cases are allocated randomly.*

Two other conditions were present in 5 of the 6 pathways: lack of challenges recruiting and retaining and lack of out-of-pocket expenses. For most activistas who achieved a high change in HIV status knowledge, their supervisors actively worked to retain activistas by ensuring that they had access to follow-up trainings and the opportunity to discuss job stress and complex beneficiaries. In CBOs 1 and 5 that had low percentage changes in HIV status knowledge, activista retention was inhibited by dissatisfaction with the activista stipend. In fact, two-thirds of activistas were unsatisfied with their stipend amount.

#### Activista Support and Experience

Five of the 6 pathways had at least 1 condition that reflected activista skills and external support: level of supportive supervision, quality of care team meetings, low supervision ratio, long training duration, and/or work experience (≥12 months). These pathways had a high amount of redundancy between conditions that ensure activistas were well equipped and well supported, which explains why most activistas were able to spend less time per case and still achieve desirable outcomes. Supportive supervision was the most effective when activista chefes met with each activista at least twice per week. Activista chefes traveled with all activistas periodically to:

*know what is happening in the communities with the activistas, know the situations and look for joint solutions, [and] verify that the beneficiaries are receiving the services required from the activistas.*

Low supervision ratios were not necessary for high levels of supportive supervision, but low ratios helped to ensure activista chefes and supervisors had more long-term time and energy to assist with complex cases.

Further, these results demonstrate alternative ways to support activistas, which may be useful to CBOs that have limited resources. For example, in Pathway 1, activista support occurred through training and high-quality and weekly care team meetings, where activistas shared experiences, discussed challenges, and created case plans. In Pathway 2, activista support occurred through training and because activistas had 2 or more years of case management experience. If a CBO has difficulty implementing regular care team meetings, hiring highly experienced activistas could provide an alternative. Similarly, training provided another layer of redundancy to activista support. Although the number of training days proved to be less important, the most effective trainings were those that employed multiple training activities (e.g., lectures, role playing, tests) and covered topics such as how to refer beneficiaries to health services, techniques to encourage ART retention, and how to cope with work stress. Overall, the presence of activista support conditions (e.g., quality of care team meetings, supervision ratio, supportive supervision) in multiple pathways may reflect how these conditions, in combination with other conditions (e.g., quality care team meetings and low supervision ratios enable activistas to discuss difficult cases, which could help alleviate challenges of case complexity, for example), provided activistas with the resources needed to effectively manage their cases.

Results demonstrated alternative ways to support activistas, which may be useful to CBOs that have limited resources.

#### Complex Cases and Activista Preparation

The first 3 pathways show how activistas who had a high percentage of complex cases that require more time still improved HIV status knowledge. The presence of high-quality team meetings, adequate training, and formal and thoughtful case assignment procedures prepared these activistas well to manage complex cases. Finally, the presence of more time per case in Pathway 2 and less time per case in Pathway 1 may mean that this condition is not particularly important; activistas can still achieve a high percentage change in HIV known status regardless of how much time they spend with each household. Some activistas spent more time per case because they managed a higher proportion of complex cases; others spent less time per case because they had fewer complex cases or because their work experience or training meant that they strategized their cases to maximize efficiency. For example, 1 activista stated:

*I first finalize my work at home and then plan my work, and I do this on a weekly basis.*

### Outcome 2: Percentage of HIV Status Known

The second outcome investigated was the percentage of beneficiaries with their HIV status known at the time of the last assessment. Five pathways led to a high percentage of beneficiaries with HIV status known (Figure 2), each with 4–6 conditions. This solution had a consistency of 0.87 and coverage of 0.42, which are acceptable values for QCA.^34^ This solution described activistas from CBOs 2, 3, 4, and 6. No conditions were necessary or sufficient; instead, the combinations of conditions had the most important influence on the outcome.

Five pathways led to a high percentage of beneficiaries with HIV status known, each with 4–6 conditions.

#### Activistas With More Work Experience

Pathways 1, 2, and 3 shared 4 conditions: work experience ≥12 months, complex cases <10%, lack of out-of-pocket costs, and less time per case. The presence of work experience ≥12 months combined with complex cases <10% is one of the main reasons why activistas were able to spend less time with each household (approximately 25 minutes or less, on average). In these first 3 pathways, activistas worked efficiently and had lower risk of becoming burned out, allowing them to provide more effective case management services to beneficiaries. Additionally, these activistas had minimal challenges: lack of out-of-pocket costs and complex cases <10% meant that activistas had the resources to complete their work and did not have many cases that required more time. For activistas who did not have high percentages of beneficiaries with HIV status known, the presence of out-of-pocket costs could be a reason. These activistas spent their own money for transport, buying food for beneficiaries, buying airtime. If activistas have access to more resources, their effectiveness may improve.

For Pathway 1, the high supervision ratio meant that the ratios of activistas to activista chefe and of activista chefe to supervisor were higher (10:1 and 5:1, respectively). However, since these activistas had more experience and fewer case challenges, the supervision ratio did not negatively affect their case management effectiveness. In Pathway 2, activistas had caseloads of 50 or fewer cases and underwent significant training (>10 days); these conditions contributed to activistas’ preparedness and energy for effective case management. In Pathway 3, short training duration reflects fewer than 5 training days. Most trainings, regardless of duration, still covered important case management topics (e.g., goal setting, common challenges). Activistas requested additional training on topics including HIV testing, dealing with sensitive cases, and family planning.

#### Activistas With Less Work Experience

Pathways 4 and 5 differed from the first 3 pathways mainly because they had work experience ≤11 months. These pathways demonstrate 2 alternative combinations of conditions that are sufficient to overcome an activista’s lack of work experience. Notably, when an activista had less than 11 months of experience, it was essential that the activista had a caseload of 50 or fewer cases and that the activista attended weekly care team meetings that were comprehensive and addressed care issues beyond paperwork. For activistas managing more cases, especially those with less experience, external support and being well networked were important. Although activista chefes and supervisors identified the “ideal” caseload to be approximately 50 cases, this is effectively the maximum number of cases an activista should manage. For this dataset, activistas faced difficulty completing their case work when their caseload exceeded 50 cases, especially since they were expected to work only 20 hours weekly.

Although these activistas had less work experience, they received support that ensured they managed cases effectively. High-quality and weekly care team meetings with near-perfect activista attendance were essential to effective case management. In the highest-quality meetings, activistas were:

*… Presenting the work [and] questions we have to the activista chefe who accompanies us to the beneficiaries’ houses [and] checking whether the forms have been filled well.*

Support in developing weekly plans and sharing of information enabled activistas to gain important skills to manage difficult cases and was a valuable source of accountability for activista preparedness. In contrast, activistas who did not attend or did not have access to high-quality care team meetings described the meetings as unhelpful where the focus was solely for correcting forms, and this was associated with lower percentages of beneficiaries with HIV status known.

### Analysis of the Negation of the Outcomes

The analysis of conditions that do not produce a high percentage change in knowledge of HIV status (i.e., negation of Outcome 1) did not produce any results. The analysis of negation of the Outcome 2 identified 1 pathway that led to a high percentage of beneficiaries with HIV status unknown (i.e., unknown or not revealed) (Figure 3). In-set membership was when the percentage of an activista’s beneficiaries with HIV status unknown was greater than or equal to 95%, and out-of-set membership was when the percentage of an activista’s beneficiaries with HIV status unknown was less than or equal to 75%. This pathway described activistas from CBOs 5 and 6 who had higher percentages of beneficiaries with unknown HIV status than the other CBOs. The pathway had 5 conditions: challenges recruiting and retaining, lack of ideal caseload, lack of supportive supervision, less time per case, and complex cases ≥10%. The solution consistency was 0.80 and the coverage was 0.09. No conditions were necessary or sufficient; instead, the combinations of conditions had the most important influence on the outcome.

The analysis of negation of Outcome 1 did not produce any results, while that for Outcome 2 identified 1 pathway.

#### Activista Retention

Challenges recruiting and retaining activistas had a negative influence on the percentage of beneficiaries with unknown HIV status. Activista chefes and supervisors attributed these challenges to low subsidies (i.e., activista salaries), delays in subsidy payment, lack of motivation, high caseloads, and lack of job preparedness. For example, a supervisor stated:

*Activistas leave because of the low subsidy or because of the workload in terms of caseloads.*

Activista retention challenges led to higher activista turnover, which may have negatively affected case management.

#### Activista Overwork

The lack of ideal caseload means that activistas who had a high percentage of beneficiaries with unknown HIV status also had a caseload that was above the ideal/maximum caseload (n=50, based on the average number of cases that activista chefes and supervisors said an activista should ideally manage). The highest caseload cited was 106 clients. Too many cases meant that an activista had too much work and could not dedicate adequate time to each beneficiary, leading to an inability to know the HIV status of each. In contrast, activista chefes whose activistas had low percentages of beneficiaries with unknown HIV status aimed to assign activistas only the ideal caseload to:

*… Not overload [them] with work [since] increasing the number [would] make it difficult for the activista to cover all the families.*

In addition to too many cases, activistas with high percentages of beneficiaries with HIV status unknown additionally managed a higher percentage of complex cases, meaning they were often unable to spend as much time employing strategies to learn the HIV status of their remaining clients. Similarly, too many cases meant activistas spent less time per case, with some activistas spending as few as 15 minutes per household.

#### Inadequate Activista Support

Activistas with high percentages of beneficiaries with unknown HIV status also lacked supportive supervision. For example, regarding the purpose of the meetings with their activista chefe, an activista stated:

*The activista chefe corrects the filled forms and we sign the central registry form.*

In contrast, activistas who received highly supportive supervision discussed their difficult cases and created goals and plans during these meetings. For example, an activista stated:

*I develop a plan, and if someone has abandoned ART treatment, that is where I need the activista chefe so that we can work together in this case.*

Another activista summarized the tasks accomplished during these meetings as:

*Talking about challenges, presenting the filled templates and being corrected and taught how to fill them correctly, undertaking simulations of the daily activities, weekly reporting, and exchange of experience.*

## DISCUSSION

This study aimed to provide actionable evidence to USAID and implementing organizations for strengthening their OVC programs. Previous studies have hypothesized ways to improve HIV case management, but there has been insufficient evaluation of the impact of these hypotheses on knowledge of HIV status, as well as their collective impact. The main findings from this study demonstrate the importance of strengthening multiple aspects of case management simultaneously, which may require efforts from both program funders and implementing organizations.

This study demonstrated the importance of strengthening multiple aspects of case management simultaneously.

To achieve a high percentage change in knowledge of HIV status and a high percentage of beneficiaries with HIV status known, the implementing organizations working in a context similar to this study may select any of the identified pathways and work on ensuring the presence of each of the conditions in the pathway. The selection of the pathway may depend on the resources that are available and the timeframe that each organization has to work with. Thus, changing and implementing the procedures for assigning cases may be more time consuming than changing the caseload. Although we acknowledge that the interventions should focus on modifying all conditions in the selected pathway collectively, since the pathways as a whole were identified to influence the outcomes (as opposed to individual conditions), we would like to share several key recommendations regarding those conditions that were present in multiple pathways. First, it is important for programs to implement formal protocols that assign cases based especially on caseload, complexity, and activista skills. To our knowledge, how cases are assigned has rarely been mentioned in the literature, let alone tied to HIV program performance. Potentially, the biggest impact of formal protocols to assign cases was that they resulted in strategized case management (i.e., more experienced activistas managed the more complex cases) and reduced overwork. Caseload was important for the 2 positive HIV program performance outcomes investigated, so formal protocols could help to maintain ideal activista caseloads. For all activistas to have adequate time and energy to manage their cases well, it is important that they do not manage more than 50 cases and manage a very low percentage of complex cases. Other programs have identified the maximum to be even lower (e.g., 30 cases),^18^^,^^27^ and programming is increasingly shifting focus to highly vulnerable populations that present more complex cases^4^; therefore, the maximum should depend on how many hours an activista is expected to work weekly, their previous work experience, and how many complex cases they manage. This is particularly important for activistas who have less prior work experience, since case manager expertise has been linked to improved HIV program performance.^9^^,^^17^ Implementing organizations and program funders may also consider increasing material resources provided to activistas because stipend increases may improve satisfaction and reduce activista turnover.

Activistas with high percentage changes in knowledge of HIV status generally did not have out-of-pocket expenses. This finding is also supported by literature that suggests case workers are more effective when they have access to all resources essential to basic care.^20^^,^^21^ Adequate care resources and comprehensive care plans have also been shown to be important for treatment program effectiveness for people living with HIV.^11^^,^^20^^,^^41^ Program funders could consider expanding HIV program budgets to account for activista reimbursement for transportation and airtime (i.e., cell phone service used for case management). HIV treatment is also often inhibited by the lack of beneficiary access to food, so many activistas mentioned that they purchased food for beneficiaries, resulting in out-of-pocket expenses. HIV program performance outcomes could further improve if program funders accounted for food for medication provision, which remains an unmet need.^9^^,^^10^

HIV program performance may also improve if implementing organizations can provide activistas with more than 1 form of high-quality support, especially to activistas with more complex cases and/or less work experience. Support can occur through regular trainings, weekly care team meetings, and weekly individual meetings with activista chefes. In particular, high-quality care team meetings were important for activistas who had less work experience since the meetings provided informal training and the opportunity to discuss challenging cases. Campbell et al.^15^ highlight the importance of social spaces for dialogue and critical thinking around HIV case management; regular team meetings can create this space and improve care team networking.^5^^,^^6^ Regular meetings with activista chefes could help activistas to develop and be held accountable to comprehensive case management plans, which were often missing for activistas with a high percentage of beneficiaries with HIV status unknown. These recommendations are supported by other studies that suggest that external support structures and the competency of management are critical to improve HIV outcomes.^14^^,^^16^

HIV program performance may improve if activistas receive more than 1 form of high-quality support, increased stipends, and adequate training.

Finally, programs could also implement protocols that seek to improve activista retention. Activista retention challenges were a major factor that inhibited knowledge of HIV status, often due to dissatisfaction with stipends and overwork. Dissatisfaction, low motivation, and emotional stress, have all been demonstrated to inhibit HIV outcomes.^14^^,^^15^ Activista retention challenges could be mitigated by increasing activista stipends and ensuring that they receive adequate training and supervision support.

### Limitations

Work satisfaction, salary amount, care team networking, and strength of wider referral network were domain (constant) conditions, and therefore their influences on the outcomes could not be analyzed, presenting an opportunity for future research. Although all activistas, activista chefes, and supervisors were highly or somewhat satisfied, programs could potentially improve satisfaction by increasing stipends and by using nonmonetary incentives to recognize good work. Nonmonetary incentives in other HIV treatment programs have helped to avoid demoralization and thus improve program performance.^14^ Respondents indicated that care team networking and the strength of wider referral network were strong. Literature highlights the importance of case worker connectedness to share challenges^5^^,^^6^^,^^14^^,^^15^ and of connections to additional services to ensure that beneficiaries receive comprehensive, whole-person care.^9^^,^^18^^,^^20^^,^^21^^,^^42^ The influence of care team networking and referral networks is an important area for future research. Similarly, since CBOs all followed similar protocols for case management, the influence of specific care methods could not be investigated. For example, program performance may also be improved by increasing the mobility of care, such as using home-based rapid testing^22^^,^^42^ or performing follow-up consultations by phone.^9^^,^^18^^,^^21^ Future research that compares programs that employ different HIV testing methods and follow-ups and have different levels of care mobility could generate greater insight into program optimization.

Case management attributes are not the only influencers of knowledge of HIV status; however, these factors were the focus of this study to identify actionable recommendations. Program performance may improve even more if the following individual-level factors can be addressed, which have been demonstrated to impact HIV outcomes: stigma,^17^^,^^43^ gender,^22^ urbanicity,^22^^,^^44^ beneficiary income,^11^^,^^22^ beneficiary education,^22^^,^^23^ and HIV testing method.^22^^,^^42^ We were also unable to consider different clinical and community-level factors that affect HIV outcomes, and these factors could potentially further improve HIV program performance outcomes. The following factors have been suggested in the literature to improve case management effectiveness and warrant further study: actual and perceived health service quality,^17^ distance to functioning health care facility,^5^^,^^6^^,^^20^^,^^23^ effectiveness of family support plans,^11^ new testing strategies (such as HIV self-testing, a focus on testing index cases of adults with HIV, and the availability of community-based testing), availability of HIV testing kits,^17^ and buy-in for HIV case management programs from the local community^15^ and district and central governments and nongovernmental organizations.^6^^,^^15^ Future research should seek to understand the intersection of case management attributes, beneficiary demographics, and clinical and community-level factors.

Lastly, this study is based on findings from 6 CBOs that were not selected randomly. Although we selected activistas within each CBO using simple random sampling, the results of the study may not be generalizable to other CBOs that are a part of the COVida project. Furthermore, this study relied primarily upon interviews with activistas and their managers to understand the important case management attributes. Although activistas estimated the amount of time they spent with each beneficiary and working for COVida per week, researcher observations could be important to better understand the relationship between time spent per case and HIV program performance outcomes. Similarly, study respondents described the importance of correctly and regularly completing case management paperwork, but paperwork completion itself was not a factor explicitly evaluated. Paperwork completion, follow-up phone calls, and other routine activista work could be included in a future study to identify additional ways to optimize HIV program efficiency and effectiveness.

## CONCLUSION

QCA was used to investigate the combinations of modifiable attributes of HIV case management programs to understand how to improve knowledge of HIV status. Two outcomes were identified to measure case management effectiveness: percentage change in knowledge of HIV status, and percentage of beneficiaries with HIV status known at the time of the last assessment. We identified 6 pathways for the first and 5 pathways for the second outcome of interest. Each pathway demonstrates an alternative combination of conditions that positively influences the outcome. Implementing partners working in similar contexts as this study may select any of the pathways, based on their available resources, to improve the outcome since each pathway is equally sufficient in achieving the outcome. Overall, based on the presence of some of the factors in multiple pathways to improve knowledge of HIV status, we suggest that implementing organizations, donors, and governments focus on several key recommendations. Implementing organizations could implement a formal process to assign cases based on complexity and caseload to alleviate overwork. Implementing organizations could also aim to increase the level of support to activistas, with activistas having adequate external support such as high-quality care team meetings, direct managers who meet with activistas weekly to assist with challenges, and/or low supervision ratios so that managers are available and not overworked. More program funding may be required to meet the need for increased activista support at current program target levels. We also suggest that, when possible, implementing organizations hire experienced activistas and provide all activistas with regular follow-up trainings so they have the tools to address challenging cases and complicated issues. This study builds theory on the important tenets of an effective case management program and has the potential to improve knowledge of HIV status for OVC and other vulnerable populations.

## Supplementary Material

20-00311-Allen-Supplement.pdf

## Translation

**En português**

**Analise Qualitativa de Comparação dos Determinantes Chave do Estado de Conhecimento do HIV nas Crianças Órfãos e Vulneráveis em Moçambique**

**Constatações Principais**

- Usando uma análise de comparação com uma “fuzzy set” identificamos combinações de factores modificáveis que os programas de HIV que apoiam órfãos, crianças vulneráveis e as suas famílias podem agir para aumentar a proporção de beneficiários que conhecem seu estado sorológico.

**Implicações Principais**

Para melhorar o estado do conhecimento do HIV, os programas devem considerar a implementação de um processo formal para:

- Atribuir os casos considerando a complexidade do caso e o número de casos para reduzir o excesso de trabalho.
- Fornecer apoio de supervisão aos activistas
- Contratar activistas experientes
- Formar activistas para resolver os casos desafiadores
- Reembolsar activistas por despesas relacionado com o trabalho

**SINOPSE**

Em Moçambique, mais de um milhão das crianças vivem com HIV ou são vulneráveis devido ao HIV. Respondendo a esta crise, o Plano de Emergência do Presidente dos Estados Unidos para o Alívio da SIDA financia programas que apoiam as crianças órfãos e vulneráveis afectadas pelo HIV e suas famílias. Esses programas retêm assistentes sociais, conhecidos como activistas, que fornecem e encaminham os beneficiários aos serviços para aumentar o conhecimento dos beneficiários sobre seu estado de HIV e para melhorar a retenção aos cuidados de saúde dos que vivem com HIV. Para melhorar a eficácia do programa, as organizações de implementação precisam entender como os diferentes atributos de gestão de caso afectam os resultados dos beneficiários. Usamos uma Analise de Comparação Qualitativa com uma série difusa (QCA) baseada em 119 entrevistas para identificar as combinações de atributos de gestão de casos que levam para (1) maior conhecimento do estado de HIV e (2) altas percentagens de beneficiários com o estado de HIV conhecido. Identificamos seis vias para o primeiro resultado e cinco vias para o segundo resultado. Cada caminho demonstra uma combinação alternativa de condições que influenciam positivamente o resultado e é igualmente suficiente para atingir o resultado. Para melhorar o conhecimento do estado do HIV, os programas em um contexto semelhante ao deste estudo podem selecionar qualquer uma das vias identificadas com base em seus recursos existentes e trabalhar para garantir a presença de cada uma das condições na via. No geral, com base na presença de alguns dos factores em vias múltiplas, concluímos que para melhorar o conhecimento do estado do HIV é importante que os programas implementem um processo formal para designar os casos, considerando a complexidade do caso e o número de casos existentes para reduzir o excesso de trabalho; fornecer aos activistas apoio externo como encontros semanais da equipe de atendimento, reuniões semanais de supervisão e/ou contratar activistas experimentados; fornecer a todos os activistas formacoes de e/ou reduzido rácio de supervisão; fornecer a todos os activistas formação de seguimento (refrescamento) para que tenham as ferramentas para lidar com os casos desafiadores, aumentar os recursos financeiros oferecidos aos activistas e reembolsar qualquer despesas relacionadas com o trabalho.

## Notes

**Peer Reviewed**

## References

[1] United Nations Children’s Fund (UNICEF). *For Every Child, End AIDS-Seventh Stocktaking Report*. UNICEF; 2016. Accessed June 6, 2020. https://data.unicef.org/wp-content/uploads/2016/12/HIV-and-AIDS-2016-Seventh-Stocktaking-Report.pdf

[2] Joint United Nations Programme on HIV/AIDS (UNAIDS). *Fact Sheet—Global AIDS update 2019*. UNAIDS; 2020. Accessed August 21, 2020. https://www.unaids.org/sites/default/files/media_asset/UNAIDS_FactSheet_en.pdf

[3] US President’s Emergency Plan for AIDS Relief (PEPFAR). *2019 PEPFAR Latest Global Results*. PEPFAR; 2019. Accessed June 6, 2020. https://www.state.gov/wp-content/uploads/2019/11/PEPFAR-Latest-Results_WAD_2019.pdf

[4] US President’s Emergency Plan for AIDS Relief (PEPFAR). *Mozambique Country Operational Plan (COP/ROP) 2017 Strategic Direction Summary*. PEPFAR; 2018. https://www.state.gov/wp-content/uploads/2019/08/Mozambique-19.pdf

[5] Chernesky RH, Grube B. Examining the HIV/AIDS case management process. Health Soc Work. 2000;25(4):243–253. 10.1093/hsw/25.4.243. 11103697

[6] Boudreau ME, Fisher CM. Providing effective medical and case management services to HIV-infected youth preparing to transition to adult care. J Assoc Nurses AIDS Care. 2012;23(4):318–328. 10.1016/j.jana.2011.06.003. 21820326

[7] Katz MH, Cunningham WE, Fleishman JA, et al. Effect of case management on unmet needs and utilization of medical care and medications among HIV-infected persons. Ann Intern Med. 2001;135(8_Part_1):557–565. 10.7326/0003-4819-135-8_Part_1-200110160-00006. 11601927

[8] London AS, Leblanc AJ, Aneshensel CS. The integration of informal care, case management and community-based services for persons with HIV/AIDS. AIDS Care. 1998;10(4):481–503. 10.1080/09540129850124019. 9828968

[9] Horwood C, Butler L, Barker P, et al. A continuous quality improvement intervention to improve the effectiveness of community health workers providing care to mothers and children: a cluster randomised controlled trial in South Africa. Hum Resour Health. 2017;15(1):39. 10.1186/s12960-017-0210-7. 28610590 PMC5470211

[10] Katz MH, Cunningham WE, Mor V, et al. Prevalence and predictors of unmet need for supportive services among HIV-infected persons: impact of case management. Med Care. 2000;38(1):58–69. 10.1097/00005650-200001000-00007. 10630720

[11] Richter LM, Sherr L, Adato M, et al. Strengthening families to support children affected by HIV and AIDS. AIDS Care. 2009;21 Suppl 1(S1):3–12. 10.1080/09540120902923121. 22380973 PMC2903779

[12] King E, De Silva M, Stein A, Patel V. Interventions for improving the psychosocial well-being of children affected by HIV and AIDS. Cochrane HIV/AIDS Group, ed. Cochrane Database Syst Rev. 2009;2009(2):CD006733. 10.1002/14651858.CD006733.pub2. 19370650 PMC7387107

[13] Heikens GT, Bunn J, Amadi B, et al; Blantyre Working Group. Case management of HIV-infected severely malnourished children: challenges in the area of highest prevalence. Lancet. 2008;371(9620):1305–1307. 10.1016/S0140-6736(08)60565-6. 18406865

[14] Nair Y, Campbell C. Building partnerships to support community-led HIV/AIDS management: a case study from rural South Africa. Afr J AIDS Res. 2008;7(1):45–53. 10.2989/AJAR.2008.7.1.6.434. 25871271

[15] Campbell C, Nair Y, Maimane S. Building contexts that support effective community responses to HIV/AIDS: a South African case study. Am J Community Psychol. 2007;39(3–4):347–363. 10.1007/s10464-007-9116-1. 17447133

[16] Foster AA, Makukula MK, Moore C, et al. Strengthening and institutionalizing the leadership and management role of frontline nurses to advance universal health coverage in Zambia. Glob Health Sci Pract. 2018;6(4):736–746. 10.9745/GHSP-D-18-00067. 30591579 PMC6370361

[17] Martin C, Masote M, Hatcher A, Black V, Venter WDF, Scorgie F. HIV testing in the critical care setting: views of patients, family members and health providers from urban South Africa. AIDS Care. 2015;27(5):581–586. 10.1080/09540121.2014.987104. 25483875

[18] Grabbe KL, Menzies N, Taegtmeyer M, et al. Increasing access to HIV counseling and testing through mobile services in Kenya: strategies, utilization, and cost-effectiveness. J Acquir Immune Defic Syndr. 2010;54(3):317–323. 10.1097/QAI.0b013e3181ced126. 20453819 PMC3225204

[19] Awusabo-Asarea K, Marfob C. Attitudes to and management of HIV/AIDS among health workers in Ghana: the case of Cape Coast municipality. Health Transit Rev. 1997;7 Suppl:271–280. 10169650

[20] Reif S, Golin CE, Smith SR. Barriers to accessing HIV/AIDS care in North Carolina: rural and urban differences. AIDS Care. 2005;17(5):558–565. 10.1080/09540120412331319750. 16036242

[21] Sarna A, Saraswati LR, Okal J, et al. Cell phone counseling improves retention of mothers with HIV infection in care and infant HIV testing in Kisumu, Kenya: a randomized controlled study. Glob Health Sci Pract. 2019;7(2):171–188. 10.9745/GHSP-D-18-00241. 31142546 PMC6641813

[22] Cherutich P, Kaiser R, Galbraith J, et al. Lack of knowledge of HIV status a major barrier to HIV prevention, care and treatment efforts in Kenya: results from a nationally representative study. PLoS One. 2012;7(5):e36797. 10.1371/journal.pone.0036797. 22574226 PMC3344943

[23] Wasti SP, Simkhada P, Randall J, Freeman JV, van Teijlingen E. Factors influencing adherence to antiretroviral treatment in Nepal: a mixed-methods study. PLoS One. 2012;7(5):e35547. 10.1371/journal.pone.0035547. 22563464 PMC3341373

[24] Ministério da Saúde (MISAU), Instituto Nacional de Estatística (INE), ICF. *Inquérito de 2015 Indicadores de Imunização, Malária e HIV/SIDA Em Moçambique 2015: Relatório de Indicatores Básicos (IMASIDA)*. MISAU, INE, ICF; 2015.

[25] Joint United Nations Programme on HIV/AIDS (UNAIDS). Fact sheet—World AIDS Day 2018. UNAIDS; 2018.

[26] United States Agency for International Development (USAID). *Controlling the HIV/AIDS Epidemic*. USAID; 2018. Accessed August 21, 2020. https://www.usaid.gov/sites/default/files/documents/1860/HIV-AIDS-PEPFAR_-_Sector_Briefer.pdf

[27] COVida. *Service Delivery and Support for Orphans and Vulnerable Children (COVIDA)*. COVida Quarterly Report; 2019.

[28] Saldana J. *The Coding Manual for Qualitative Researchers*. 2nd ed. Sage Publications; 2009.

[29] Ritchie J, Lewis J. *Qualitative Research Practice: A Guide for Social Science Students and Researchers*. Sage Publications; 2003.

[30] Davis A, Javernick-Will A, Cook SM. The use of qualitative comparative analysis to identify pathways to successful and failed sanitation systems. Sci Total Environ. 2019;663(1):507–517. 10.1016/j.scitotenv.2019.01.291. 30716642

[31] Opdyke A, Javernick-Will A, Koschmann M. A comparative analysis of coordination, participation, and training in post-disaster shelter projects. Sustainability. 2018;10(11):4241. 10.3390/su10114241

[32] Basurto X, Speer J. Structuring the calibration of qualitative data as sets for qualitative comparative analysis (QCA). Field Methods. 2012;24(2):155–174. 10.1177/1525822X11433998

[33] Ragin CC. *The Comparative Method: Moving Beyond Qualitative and Quantitative Strategies*. University of California Press; 1987.

[34] Ragin CC. *Redesigning Social Inquiry: Fuzzy Sets and Beyond*. University of Chicago Press; 2008.

[35] Kaminsky J, Jordan E. Qualitative comparative analysis for WASH research and practice. J Water Sanit Hyg Dev. 2017;7(2):196–208. 10.2166/washdev.2017.240

[36] Ragin CC. Fuzzy sets: calibration versus measurement. In: Collier D, Brady H, Box-Steffensmeier J, eds. *Methodology Volume of Oxford Handbook of Political Science*. Oxford University Press; 2007.

[37] Schneider CQ, Wagemann C. Standards of good practice in qualitative comparative analysis (QCA) and fuzzy-sets. Comp Sociol. 2010;9(3):397–418. 10.1163/156913210X12493538729793

[38] Ragin CC, Patros T, Strand SI, Rubinson C. *User’s Guide to Fuzzy-Set/Qualitative Comparative Analysis*. Ragin; 2017. Accessed August 21, 2020. http://www.socsci.uci.edu/∼cragin/fsQCA/download/fsQCAManual.pdf

[39] Ragin CC. Set relations in social research: evaluating their consistency and coverage. Polit Anal. 2006;14(3):291–310. 10.1093/pan/mpj019

[40] Rihoux B, Ragin CC. *Configurational Comparative Methods: Qualitative Comparative Analysis (QCA) and Related Techniques*. Sage Publications, Inc.; 2009.

[41] Poteat T, Malik M, Scheim A, Elliott A. HIV prevention among transgender populations: knowledge gaps and evidence for action. Curr HIV/AIDS Rep. 2017;14(4):141–152. 10.1007/s11904-017-0360-1. 28752285 PMC5896563

[42] Hutchinson AB, Branson BM, Kim A, Farnham PG. A meta-analysis of the effectiveness of alternative HIV counseling and testing methods to increase knowledge of HIV status. AIDS. 2006;20(12):1597–1604. 10.1097/01.aids.0000238405.93249.16. 16868440

[43] Emlet CA. A comparison of HIV stigma and disclosure patterns between older and younger adults living with HIV/AIDS. AIDS Patient Care STDS. 2006;20(5):350–358. 10.1089/apc.2006.20.350. 16706709

[44] Alizadeh F, Mfitumuhoza G, Stephens J, et al. Identifying and reengaging patients lost to follow-up in rural Africa: the “horizontal” hospital-based approach in Uganda. Glob Health Sci Pract. 2019;7(1):103–115. 10.9745/GHSP-D-18-00394. 30926739 PMC6538125

