# Supplementary material for: A Qualitative Comparative Analysis of the Drivers of HIV Status Knowledge in Orphans and Vulnerable Children in Mozambique
Source: Glob Health Sci Pract. 2020 Sep 30;8(3):534–48. doi: 10.9745/GHSP-D-20-00311 (PMC7541122; doi:10.9745/GHSP-D-20-00311)
Supplement: 20-00311-Allen-Supplement.pdf [file 20-00311-Allen-Supplement.pdf]

Supplement to: Davis A, Allen E, do Nasciemento N, Chapman J, Donco R, Velthausz D. A qualitative comparative analysis of the drivers of HIV status knowledge in orphans and vulnerable children in Mozambique. *Glob Health Sci Pract*. 2020;8(3). https://doi.org/10.9745/GHSP-D-20-00311

## Supplement. Qualitative Comparative Analysis Calibration Guide

This document provides the complete guide to the calibrated outcomes and causal conditions used in the qualitative comparative analysis (QCA). As an overview of the outcome values, Figure S summarizes the percentage of beneficiaries whose HIV status changed to known, by community-based organization (CBO), and Figure S summarizes the percentage of beneficiaries whose HIV status was unknown at enrollment and last assessment, by CBO.

**Figure S1.** Proportion of beneficiaries who learned their HIV status while a COVida beneficiary, by CBO (N=1,897)

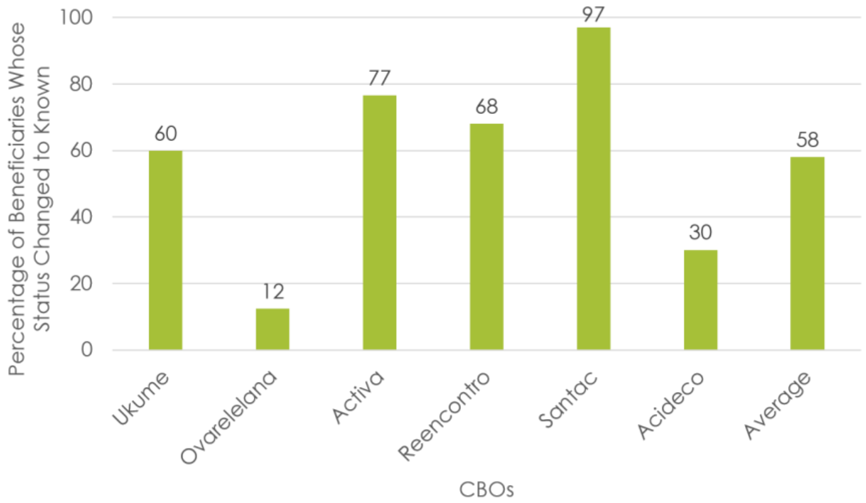


**Figure S2.** Proportion of beneficiaries with HIV unknown or unrevealed status at enrollment (N=6,029) and last assessment (N=5,146)

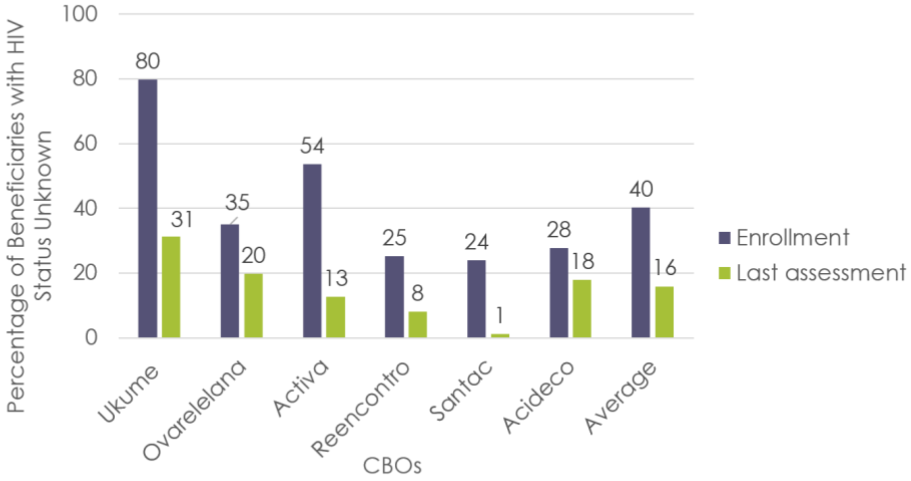

## OUTCOMES

### Outcome 1: Change in HIV Known Status

The first outcome investigated was the percentage of beneficiaries who changed their reported HIV status from unknown or not revealed to known. This outcome, referred to as “change in HIV status known” was measured as the percentage of an activista’s beneficiaries who learned their HIV status between enrollment and July 2019. These beneficiaries were enrolled before April 1, 2019, and had their HIV status recorded at least one time in addition to the time of enrollment. HIV status was considered known if the beneficiary status was HIV positive, on antiretroviral therapy (ART), not on ART (likely HIV-positive status but not receiving treatment), or test not recommended (status likely not HIV positive). HIV status was considered unknown if the beneficiary status was unknown or not revealed. A change in status was recorded when a beneficiary’s HIV status went from one of the unknown categories to one of the known categories between the time of their enrollment and the last assessment (July 2019). In-set membership was when the percentage of an activista’s beneficiaries with a change in HIV known status was greater than or equal to 75%. Out-of-set membership was when the percentage of an activista’s beneficiaries with a change in HIV known status was less than or equal to 25%. The crossover point was when the percentage of an activista’s beneficiaries with a change in HIV known status was equal to 50%. These cutoff values were determined based on breakpoints identified from the raw, plotted outcome data (Figure ). The outcome was measured quantitatively and was calibrated directly (Figure ).

**Figure S3. Raw values for the percentage of each activista's cases where HIV known status changed**

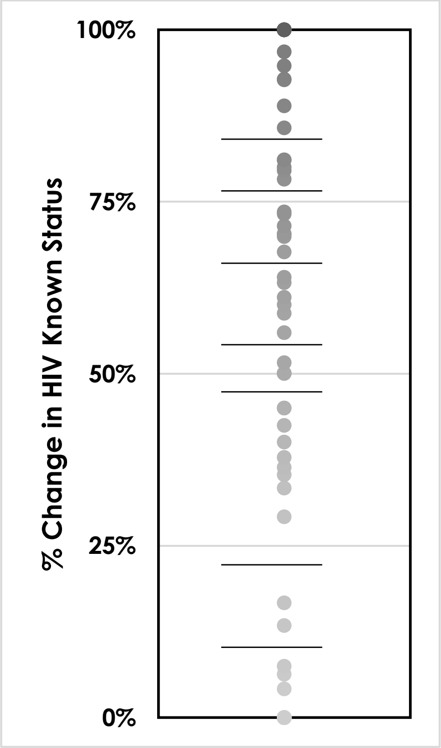


**Figure S4. Direct calibration for Outcome 1: change in HIV status known**

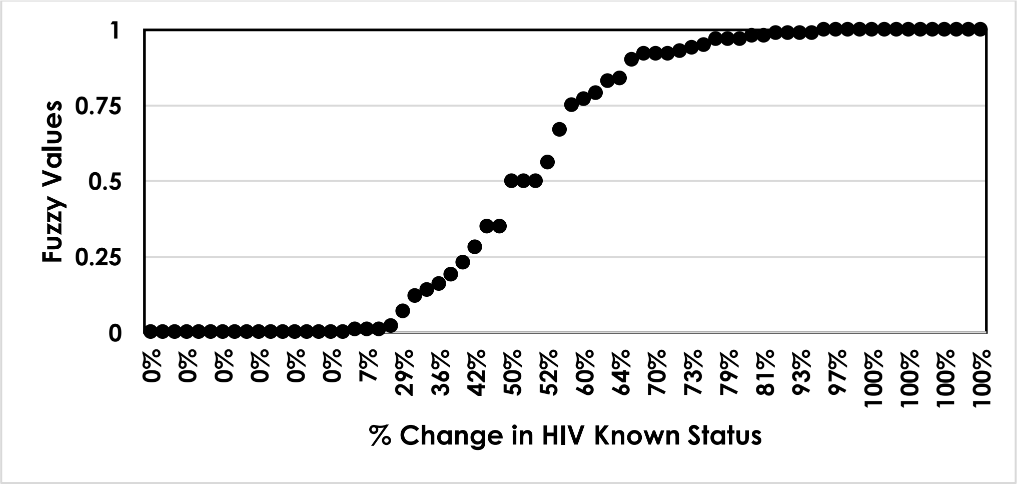


## Outcome 2: Percentage of Beneficiaries with HIV Status Known

The second outcome investigated was the percentage of beneficiaries with their HIV status known at the time of the last assessment. This outcome was measured as the percentage of beneficiaries

whose status was either HIV positive or negative (and was not unknown or not revealed) at the time of the last assessment. In-set membership was when the percentage of an activista's beneficiaries with HIV status known was greater than or equal to 95%. Out-of-set membership was when the percentage of an activista's beneficiaries with HIV status known was less than or equal to 75%. The crossover point was when the percentage of an activista's beneficiaries with HIV status known was equal to 80%. These cutoff values were determined based on breakpoints identified from the raw, plotted outcome data (

Figure S55). The outcome was measured quantitatively and was calibrated directly (

Supplement to: Davis A, Allen E, do Nasciemento N, Chapman J, Donco R, Velthausz D. A qualitative comparative analysis of the drivers of HIV status knowledge in orphans and vulnerable children in Mozambique. *Glob Health Sci Pract*. 2020;8(3). https://doi.org/10.9745/GHSP-D-20-00311

Figure 6).

**Figure S5. Raw values for the percentage of each activista's cases where HIV status was known at the last assessment**

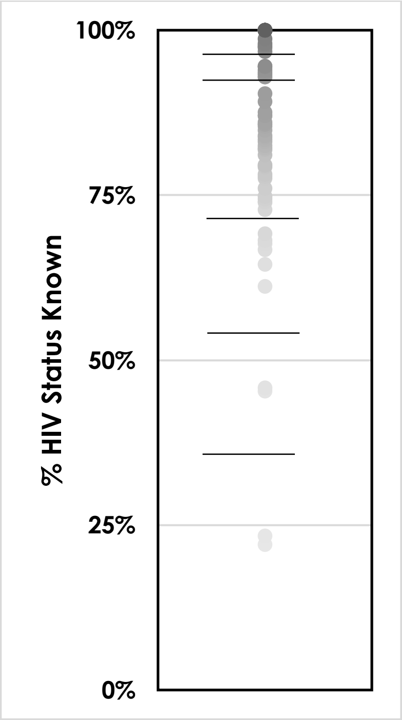

Supplement to: Davis A, Allen E, do Nasciemento N, Chapman J, Donco R, Velthausz D. A qualitative comparative analysis of the drivers of HIV status knowledge in orphans and vulnerable children in Mozambique. *Glob Health Sci Pract*. 2020;8(3). https://doi.org/10.9745/GHSP-D-20-00311

**Figure S6. Direct calibration for Outcome 2: Percentage HIV status known**

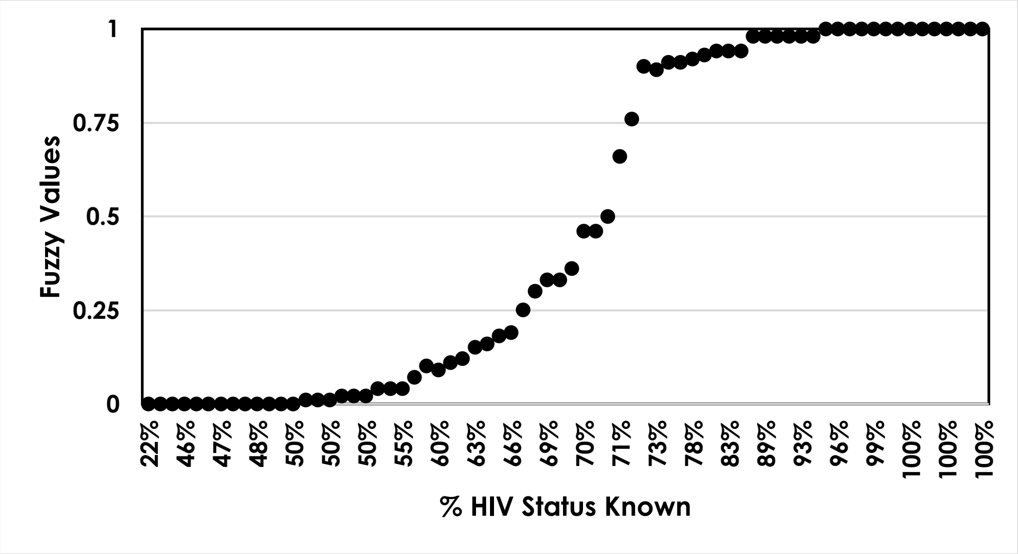


Outcome 2 Negation Analysis: Percentage of Beneficiaries with HIV Status Unknown
The third outcome investigated was the percentage of beneficiaries with their HIV status unknown at the time of the last assessment. This outcome was measured as the percentage of beneficiaries whose HIV status was unknown or not revealed. Since beneficiaries will have been enrolled for at least 3 months, it is expected that 3 months is sufficient time to discover their HIV status if case management is done well. Therefore, the variables that relate to effective case management are also expected to positively influence knowledge of HIV status during that time. In-set membership was when the percentage of an activista's beneficiaries with HIV status unknown was greater than or equal to 95%. Out-of-set membership was when the percentage of an activista's beneficiaries with HIV status known was less than or equal to 75%. The crossover point was when the percentage of an activista's beneficiaries with HIV status known was equal to 80%. These cutoff values were determined based on breakpoints identified from the raw, plotted outcome data (

Figure 7). The outcome was measured quantitatively and was calibrated directly (Figure 8).

**Figure S7.** Raw values for the percentage of each activista's cases where HIV status was unknown at the last assessment

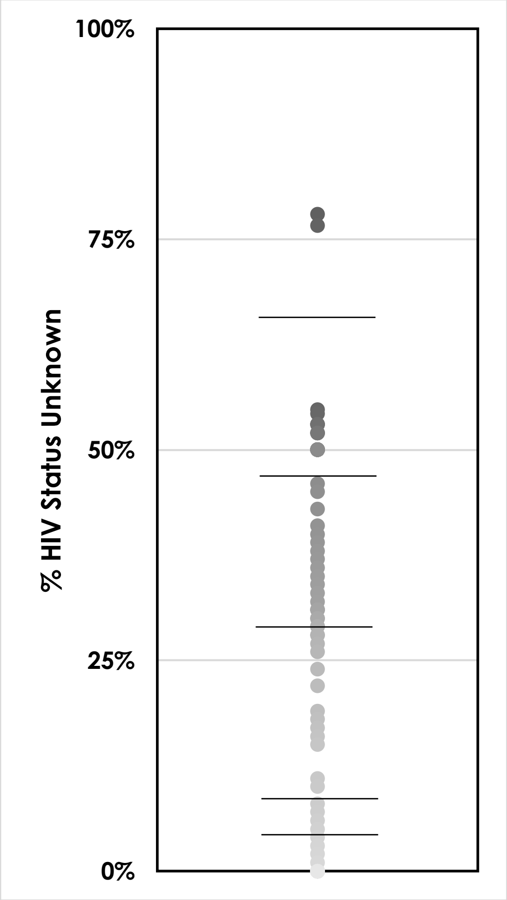


**Figure S8.** Direct calibration for Outcome 2 negation analysis: Percentage HIV status unknown

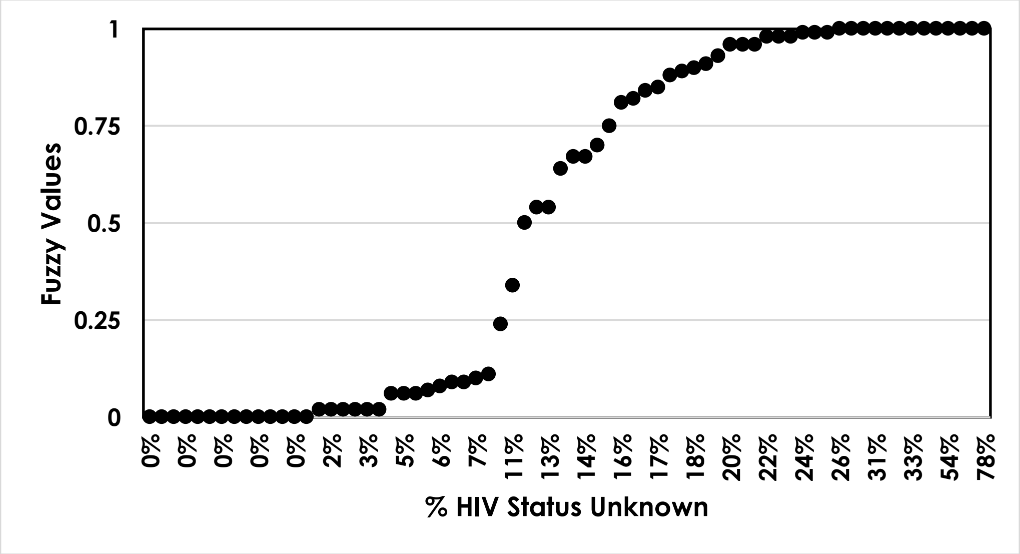

## Potential Causal Conditions

Table 1 summarizes the potential causal conditions considered in the analysis of the outcomes.

**Table S1.** Summary of all potential causal conditions influencing HIV status outcomes

| Causal Conditions | Abbreviation for fsQCA Software | Definition | Assumption for Influence on Outcomes[a] |
|---|---|---|---|
| Caseload | Caseload | The number of unique cases that the activista is currently managing | Presence (A caseload that more closely matches the ideal caseload leads to better case management.) |
| Complexity | Complexity | The percentage of an activista's caseload in which clients require more time (i.e., HIV-positive status or status not revealed) | Absence (A lower proportion of complex cases that require more time allows for better case management.) |
| Challenges in recruiting and retaining activistas | ChalRecruitRetain | Difficulties that a CBO experiences in recruiting or retaining activistas | Absence (The lack of challenges in recruiting and retaining activistas leads to better case management.) |
| How cases are assigned | HowCaseAssigned | All decisions and activities associated with how cases are assigned to an activista | Presence (Case assignment that considers more factors leads to better case management.) |
| Level of education | LevelofEducation | The number of years and type of education the activista has | Presence (Higher education leads to better case management.) |
| Level of supportive supervision | LevSuppSuper | The amount of assistance, support, and help for effective case management that an activista receives from supervisor | Presence (Highly supportive supervision leads to better case management.) |

| Causal Conditions | Abbreviation for fsQCA Software | Definition | Assumption for Influence on Outcomes[a] |
|---|---|---|---|
| Nonmonetary incentives | NonMonInc | All nonmonetary compensation that activistas receive as incentives or rewards for performing more effective case management, such as gifts, awards, certificates, or thank you letters | Presence (Nonmonetary compensation leads to better case management.) |
| Out-of-pocket costs | OutofPocket | Any expenses an activista incurs related to case management (e.g., SMS, transport) that are not paid for or reimbursed by the CBO | Absence (The lack of out-of-pocket expenses leads to better case management.) |
| Quality-of-care team meetings | QualTeamMtgs | The beneficial activities associated with routine meetings between an activista, their peers (other activistas), and their supervisors (activista chefes), and the frequency with which the meetings are held and attended by the activista | Presence (High-quality team meetings lead to better case management.) |
| Supervision ratio | SupRatio | The number of activistas assigned to each activista chefe, and the number of activista chefes assigned to each supervisor | Presence (Lower ratios lead to better case management.) |
| Task ratio | TaskRatio | The ratio of minutes an activista spends on cases to the minutes an | Presence (The more time an activista spends directly focused on cases |

| Causal Conditions | Abbreviation for fsQCA Software | Definition | Assumption for Influence on Outcomes[a] |
|---|---|---|---|
| | | activista spends on administrative tasks | leads to better case management.) |
| Time spent per case | TimeCase | The average number of minutes that an activista spends with one case | Presence or absence (The more time spent with each household could lead to better case management OR burnout and subsequently poor case management.) |
| Time spent working for COVida | TotTimeCOVida | The total number of hours per month that an activista spends on all activities related to case management and their role as an activista | Presence or absence (The more time spent managing cases could lead to better case management OR burnout and subsequently poor case management.) |
| Training | Training | All activities associated with the formal training program an activista participated in upon being hired for case management and the number of training days an activista received | Presence (More training leads to better case management) |
| Work experience | WorkExp | The maximum value of the number of years/months of COVida experience and other activista experience | Presence (More work experience leads to better case management) |

[a]To simplify the QCA, researchers often make assumptions based on case knowledge and theory of whether the presence or absence of a condition will lead to the outcome(s) of interest.[1] If there is not sufficient evidence to suggest the directionality of the condition's influence on the outcome, both the presence and the absence of the condition are analyzed. These assumptions are for Outcomes 1 and 2; the opposite of the listed assumptions applies for Outcome 2 negation.

[1] Ragin CC. *Redesigning Social Inquiry: Fuzzy Sets and Beyond*. University of Chicago Press; 2008.

**Caseload**

*Caseload* was defined as the difference between the number of unique cases (i.e., households) that the activista is currently managing and the average ideal number of cases (as identified in the survey from activista chefes and supervisors). From the survey results, most activista chefes and supervisors considered a caseload of approximately 50 cases to be ideal. Caseloads that are closer to the ideal number are hypothesized to positively influence effective case management and, therefore, a positive change in both study outcomes. *Caseload* was calibrated using the direct calibration method. The anchor points were determined based on the spread of activista chef and supervisor responses for ideal caseload (Figure S99). In-set membership was when the difference between the number of cases an activista managed and the average ideal caseload was zero (

Figure S10). Out-of-set membership was when the difference between the number of cases an activista managed and the average ideal caseload was 20. The crossover point was when the difference between the number of cases an activista managed and the average ideal caseload was 10.

**Figure S9. Raw values for the difference in the number of cases an activista currently manages and the average ideal caseload**

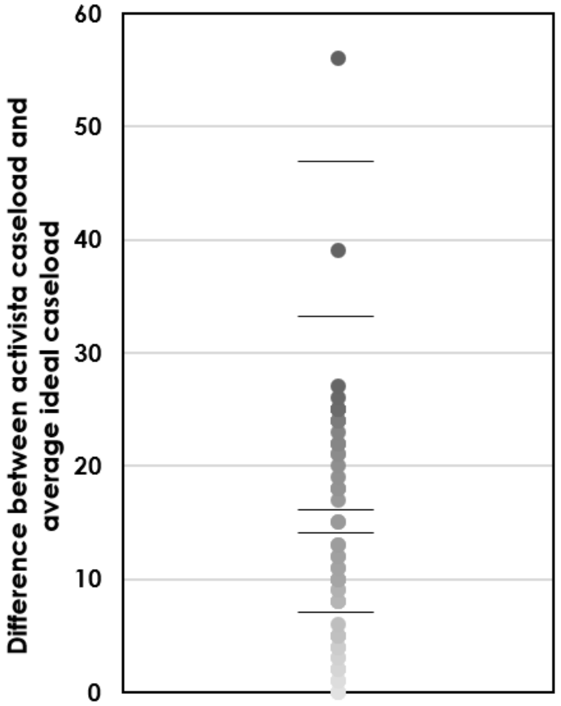

**Figure S10. Direct calibration for caseload**

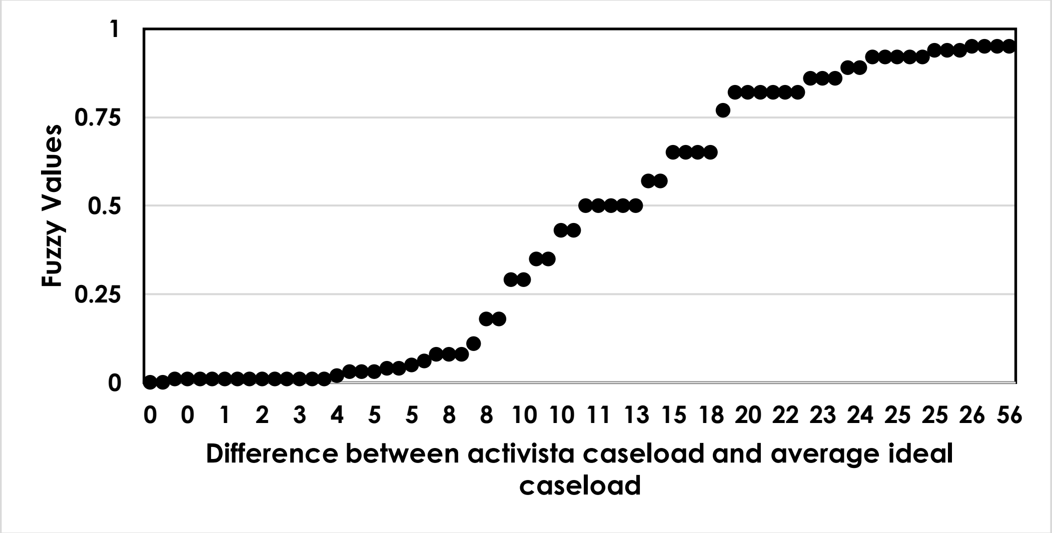


## Challenges in Recruiting and Retaining Activistas

*Challenges in recruiting and retaining activistas* was defined as difficulties that a CBO experiences in recruiting or retaining activistas. The absence of recruiting and retaining challenges was hypothesized to positively influence effective case management and, therefore, a positive change in both study outcomes. In-set membership was when the care team reported significant issues with recruiting and retaining activistas, was under-staffed, and did not have a plan to improve activista retention (Table S2). Out-of-set membership was when the entire care team did not report issues with recruiting or retaining activistas and was fully staffed.

**Table S2.** Indirect calibration for *challenges in recruiting and retaining activistas*

| Fuzzy Value | Calibration |
|---|---|
| 1 | The care team reports significant issues with recruiting and retaining activistas, is understaffed, and lacks a clear plan to recruit and retain activistas. |
| 0.67 | There are many issues with activista recruiting or retention, and activistas leave for reasons beyond the low subsidy. The care team may have plans to alleviate activista turnover, but no action has been taken. |
| 0.33 | There are some issues with activista recruiting or retention, such as activistas leaving due to low subsidies. The care team demonstrates clear actions and plans devised to alleviate activista turnover. |
| 0 | The entire care team does not report issues with recruiting or retaining activistas, is fully staffed, and has a clear plan in place to recruit and retain activistas. |

**Complexity**

*Complexity* was defined as the percentage of an activista's total caseload that were clients who required more time (i.e., clients who were living with HIV or clients whose HIV status was not revealed). Caseloads with fewer complex cases were hypothesized to positively influence effective case management and, therefore, a positive change in both study outcomes. Raw data for complexity came from HIV status at the last assessment (Figure S21). This variable was also measured and calibrated using data from beneficiaries with at least 2 assessments and from beneficiaries living with HIV only; in both of these instances, necessity scores were lower than complexity as measured relying on the last assessment; therefore, complexity was calibrated using data from the last assessment because this also accounted for more of an activista's total beneficiaries. In-set membership was when 15% or more of an activista's clients required more time (

Figure S32). Out-of-set membership was when 5% or fewer of an activista's clients required more time. The crossover point was when 10% of an activista's clients required more time.

**Figure S21.** Raw data for caseload complexity, whereby complexity was defined as the percentage of an activista's caseload who were clients who required more time (i.e., were either living with HIV or status not revealed)

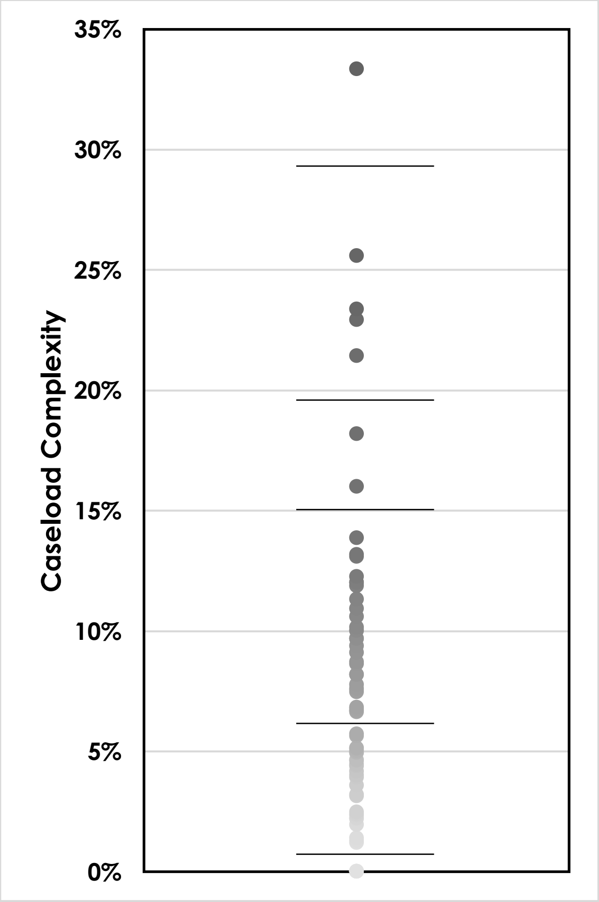

**Figure S32.** Direct calibration for *caseload complexity*

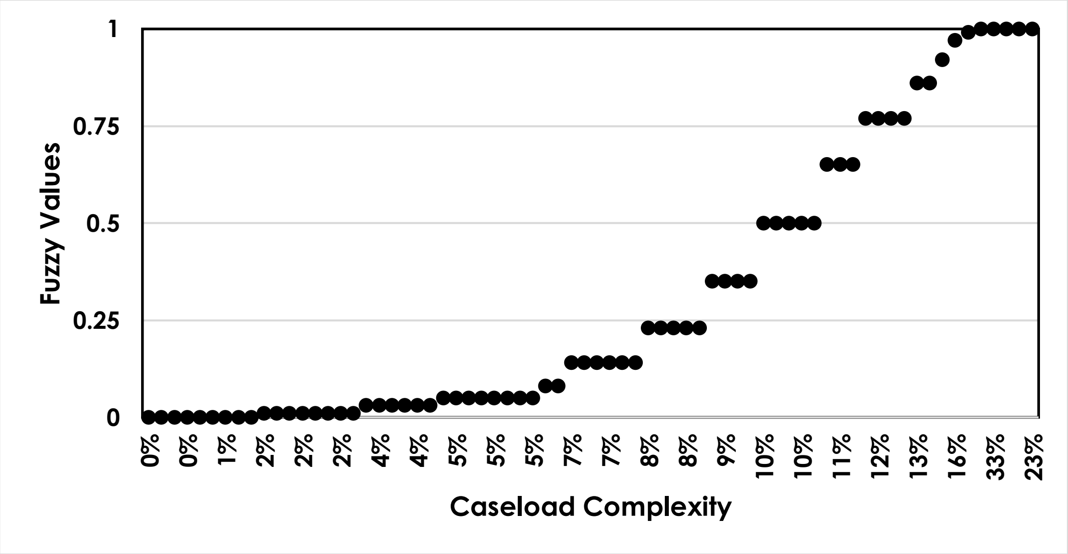


## How Cases Are Assigned

*How cases are assigned* was defined as all decisions and activities associated with how cases were assigned to an activista. A process that considered many factors, especially complexity, was hypothesized to positively influence effective case management and, therefore, a positive change in both study outcomes. In-set membership was when there was a clear, formalized process to assign cases that was always followed and ensured that cases were assigned to activistas relatively equally; this process assigned cases based on existing caseload, case complexity, activista experience and skills, and activista proximity to the case (Table 3). Out-of-set membership was when there was no formalized process to assign cases, and care team members were unsure of the process.

**Table S3.** Indirect calibration for *how cases are assigned*

| Fuzzy Value | Calibration |
|---|---|
| 1 | There is a clear, formalized process to assign cases that is always followed and ensures that cases are assigned to activistas equally. Cases are assigned based on case complexity and at least 3 of the 4 other factors: caseload, activista experience and skills, and activista proximity to household. |
| 0.67 | Cases are assigned based on at least 3 of the 4 following factors: existing caseload, activista experience and skills, and activista proximity to household. |
| 0.33 | Cases are assigned based primarily on activista proximity to the household and/or only one of the following other factors: activista experience and skills, and existing caseload. Case complexity is not considered. |
| 0 | There is no formalized process to assign cases (e.g., no factors are considered), and care team members are unsure of the process. |

## Level of Education

*Level of education* was defined as the highest level of education an activista had completed (i.e., primary, secondary, technical/professional, or university). A higher level of education was posited to influence effective case management and, therefore, a positive change in each of the 2 study outcomes. In-set membership was when an activista had some postsecondary education such as technical or professional training or university education (Table S4). Out-of-set membership was when an activista had only primary education or below. A 3-value fuzzy set was chosen for this causal condition because no activistas had any university education, and most activistas had completed some secondary education.

**Table S4.** Indirect calibration for *level of education*

| Fuzzy Value | Calibration |
|---|---|
| 1 | The activista has completed some technical or professional postsecondary education such as a vocational school or certificate program and/or has completed some university education. |
| 0.7 | The activista has completed some secondary education. |
| 0 | The activista has completed only primary education or has not had any type of formal education. |

## Level of Supportive Supervision

*Level of supportive supervision* was the amount of assistance, support, and help for effective case management that an activista received from their supervisor. Supportive supervision was posited to be critical for effective case management because it provides direction setting and oversight that are critical, particularly for work with vulnerable populations. In-set membership was when the activista met with their activista chef for 6 or more hours per week (Table 5). The activista felt comfortable voicing any issue or challenge to their activista chef and felt well supported. Additionally, the activista met regularly with their activista chefe, and their activista chefe helped with most or all of the following tasks: helped the activista set goals, developed family support plans and next steps for complex cases, provided time management guidance, reviewed case files for completion and accuracy, helped resolve challenges, provided assistance for referring clients to other services, identified knowledge gaps and training needs and provided a means to close those gaps, and accompanied the activista on home visits. Out-of-set membership occurred when the activista did not meet with their activista chef or meetings happened less than quarterly, and when the activista felt unsupported by their supervisor.

**Table S5.** Indirect calibration for *level of supportive supervision*

| Fuzzy Value | Calibration |
|---|---|
| 1 | The activista feels comfortable voicing any issue or challenge to their activista chefe and feels well-supported. Additionally, the activista meets regularly with their activista chefe (i.e., 3 or more times per week), and their meeting duration is for 6 or more hours per week. Their activista chefe helps with most or all of the following tasks: helps the activista set goals, develop family support plans and next steps for complex cases, provides time management guidance, reviews case files for completion and accuracy, helps resolve challenges, provides assistance for referring clients to other services, identifies knowledge gaps and training needs and provides a means to close those gaps, and accompanies the activista on home visits. The activista chef receives a similar level of support from their supervisor. |
| 0.67 | The activista feels supported by their activista chef. The activista meets somewhat frequently with their activista chefe (i.e., at least once a week), and their meeting duration is between 2 and 5 hours per week. Their activista chefe helps with several of the following tasks: helps the activista with time management, helps refer clients to other services, identifies activista training/skills gaps, resolves challenges, and occasionally accompanies the activista on home visits. The activista may have a suggestion for how their supervision could be more supportive. The activista chefe receives a similar level of support from their supervisor. |
| 0.33 | The activista meets somewhat infrequently with their activista chefe (i.e., once a month) and/or their meeting duration is less than 2 hours per week. Their activista chefe helps with some tasks, such as resolving challenges or reviewing case files, but this help does not occur regularly. Notably, the activista chef does not help with referring clients to other services, time management, or identifying activista training/skills gaps. The activista expresses a need for more support. The activista chefe receives a similar level of support from their supervisor. |
| 0 | The activista meets with their activista chef infrequently (i.e., once a month or less) and for less than an hour each time. The meetings exclusively cover administrative tasks (e.g., signing forms), and the activista feels unsupported by their supervisor. The activista chefe receives a similar level of support from their supervisor. |

Nonmonetary Incentives

*Nonmonetary incentives* was defined as all nonmonetary compensation that activistas received as incentives or rewards for performing more effective case management, such as gifts, awards, certificates, or thank you letters. The presence of nonmonetary incentives was hypothesized to positively influence effective case management and, therefore, a positive change in both study outcomes. In-set membership was when an activista had received one or more nonmonetary incentives (Table S6). Out-of-set membership was when the activista had not received any nonmonetary incentives.

**Table S6.** Indirect calibration for *nonmonetary incentives*

| Fuzzy Value | Calibration |
|---|---|
| 1 | The activista has received 1 or more nonmonetary incentives. |
| 0 | The activista has never received a nonmonetary incentive. |

**Out-of-Pocket Costs**

*Out-of-pocket costs* was defined as any expenses an activista incurred related to case management (e.g., SMS, transport) that was not paid for or reimbursed by the CBO. The absence of out-of-pocket costs was hypothesized to positively influence effective case management and, therefore, a positive change in both study outcomes. In-set membership was when the activista incurred weekly out-of-pocket expenses greater than or equal to 200 MT (10% of the activista salary) (Table S77). Out-of-set membership was when the activista never incurred out-of-pocket expenses. The crossover point was when the activista incurred weekly out-of-pocket expenses of 100 MT (5% of the activista salary).

**Table S7.** Indirect calibration for *out-of-pocket costs*

| Fuzzy Value | Calibration |
|---|---|
| 1 | The activista incurs weekly out-of-pocket expenses of 200 MT or greater. |
| 0.67 | The activista incurs weekly out-of-pocket expenses between 100 and 200 MT. |
| 0.33 | The activista incurs weekly out-of-pocket expenses up to (but not including) 100 MT. |
| 0 | The activista never incurs out-of-pocket expenses. |

**Quality of Care Team Meetings**

*Quality of care team meetings* was defined as the beneficial activities associated with routine meetings between an activista, their peers (other activistas), and their supervisors (activista chefes), and the frequency with which the meetings were held and attended by the activista. Typically in these meetings, activistas sign paperwork, figure out weekly/monthly plans, learn about new forms, discuss problems/issues encountered, talk about how to behave around beneficiaries, and ask questions. These activities were hypothesized to positively influence an activista's case management. In-set membership for quality-of-care team meetings was when the activista regularly attended team meetings at least weekly, and the meetings regularly included multiple activities, such as goal setting, problem resolution, expected behavior, experience sharing, check-ups on activistas' emotional well-being, follow-up on referrals, and asking questions (Table 8). Out-of-set membership was when team meetings did not occur.

**Table S8.** Indirect calibration for *quality of care team meetings*

| Fuzzy Value | Calibration |
|---|---|
| 1 | Team meetings occur regularly and frequently (i.e., weekly), and the activista attends all team meetings. Team meetings are used for goal setting and accountability, problem resolution, discussions of expected behavior, experience sharing, planning, announcements of new forms or procedures, check-ups on activistas' emotional well-being, follow-up on referrals, and asking questions. Activistas participate equally in the meetings and describe the meetings as very beneficial. |
| 0.67 | Team meetings are more frequent (i.e., usually weekly), and the activista may miss some meetings per quarter. Team meetings are usually used for planning, announcements of new forms or procedures, or for asking questions. Activistas describe the meetings as usually beneficial. |
| 0.33 | Team meetings are less frequent (i.e., monthly), and the activista attends all meetings. Team meetings are usually used for planning, announcements of new forms or procedures, or for asking questions. Activistas describe the meetings as usually beneficial. |
| 0 | Team meetings are less frequent (i.e., monthly) and/or are used only to sign paperwork. The activista misses one or more meetings per quarter. Activistas do not describe the meetings as beneficial. |

**Supervision Ratio**

*Supervision ratio* was defined as the number of activistas assigned to each activista chefe. Lower ratios were hypothesized to positively influence effective case management and, therefore, a positive change in both study outcomes. The overall *supervision ratio* condition was aggregated from 2 micro-conditions—chefe:activista ratio and supervisor:chefe ratio. Since the chefe:activista supervision ratio likely has the most direct influence on each case due to their more frequent interactions, it is likely that this supervision ratio is more influential in overall case management than that of the chefes and supervisors. Consequently, the *supervision ratio* condition used a weighted sum from the 2 micro-conditions (Table S9), where the fuzzy value for the chefe:activista ratio was weighted by 0.67, and the fuzzy value for the supervisor:chefe ratio was weighted by 0.33. In-set membership was when an activista chefe supervised 6 or fewer activistas (Table S10) and a supervisor supervised 3 or fewer chefes (Table S21). Out-of-set membership was when an activista chef supervised more than 10 activistas and a supervisor supervised more than 5 chefes.

**Table S9.** Aggregation method for *supervision ratio*

| Weighting | Micro-condition |
|---|---|
| 0.67 | Chefe:activista ratio |
| 0.33 | Supervisor:chefe ratio |

**Table S10.** Indirect calibration for *chefe:activista ratio* (micro-condition of *supervision ratio*)

| Fuzzy Value | Calibration |
|---|---|
| 1 | The chefe supervises 6 or fewer activistas. |
| 0.8 | The chefe supervises 7 activistas. |
| 0.6 | The chefe supervises 8 activistas. |
| 0.4 | The chefe supervises 9 activistas. |
| 0.2 | The chefe supervises 10 activistas. |
| 0 | The chefe supervises more than 10 activistas. |

**Table S21.** Indirect calibration for *supervisor:chefe ratio* (micro-condition of *supervision ratio*).

| Fuzzy Value | Calibration |
|---|---|
| 1 | The supervisor supervises 3 or fewer chefes. |
| 0.67 | The supervisor supervises 4 chefes. |
| 0.33 | The supervisor supervises 5 chefes. |
| 0 | The supervisor supervises more than 6 chefes. |

**Task Ratio**

*Task ratio* was defined as the ratio of the total number of hours per month that an activista spent on active case management to the total number of hours per month that an activista spent on administrative tasks (e.g., paperwork). More time spent directly on case management was hypothesized to positively influence effective case management and, therefore, a positive change in both study outcomes. *Task ratio* was calibrated directly (

Figure S43). In-set membership was when the ratio of time spent on case conferencing versus administrative tasks was greater than or equal to 4, demonstrating that the activista spent a majority of their time focused on their casework (

Figure S54). Out-of-set membership was when the ratio of time spent on case conferencing versus administrative tasks was less than or equal to 1, demonstrating that the activista spent a significant amount of time consumed with administrative tasks. The crossover point was when the ratio of time spent on case conferencing versus administrative tasks was 2.5.

**Figure S43.** Raw data for *task ratio*

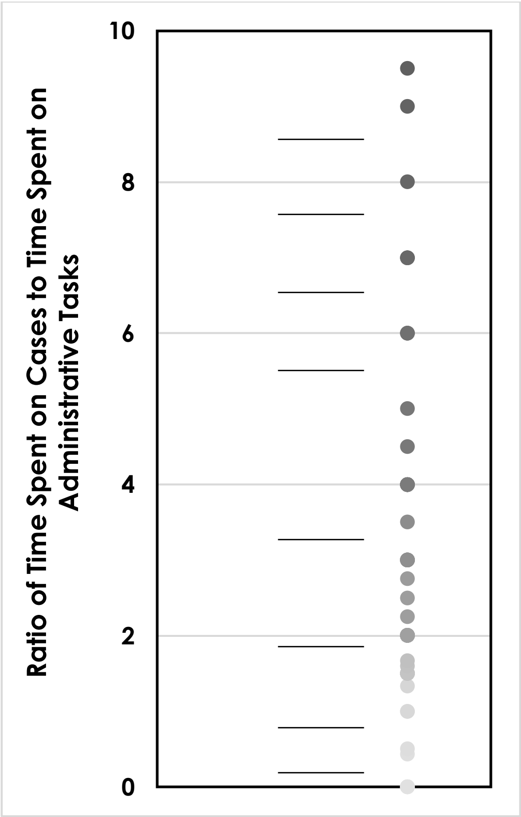

**Figure S54.** Direct calibration for *task ratio*

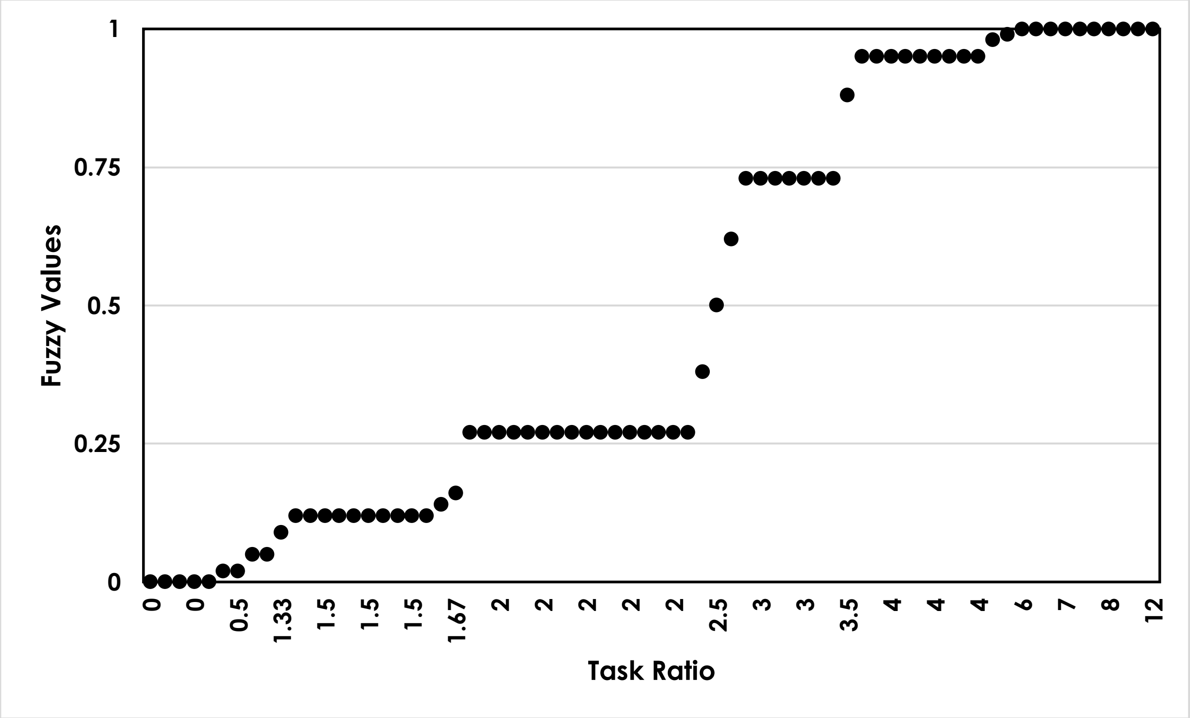


## Time Spent Per Case

*Time spent per case* was defined as the average number of minutes that an activista spent with one case. A higher number of minutes spent with each household could either positively or negatively influence the outcomes. It is possible that the presence of this condition means that an activista is able to be more thorough and has enough time to address all the needs and concerns of their clients on each visit. It is also possible that the more time an activista spends with each household, the more likely they are to burn out and, therefore, offer ineffective case management. In-set membership was when an activista spent 100 or more minutes for one case, on average (Table S32). Out-of-set membership was when an activista spent 30 or fewer minutes for 1 case, on average.

**Table S32.** Indirect calibration for *time spent per case*

| Fuzzy Value | Calibration |
|---|---|
| 1 | Activista spends 100 minutes or more with each household, on average. |
| 0.67 | Activista spends between 60 and 99 minutes with each household, on average. |
| 0.33 | Activista spends between 30 and 59 minutes with each household, on average. |
| 0 | Activista spends 30 minutes or less with each household, on average. |

## Time Spent Working for COVida

*Time spent working for COVida* was defined as the total number of hours per month that an activista spent on all activities related to case management and their role as an activista. A greater amount of time spent working for COVida per week was hypothesized to positively influence effective case management and, therefore, a positive change in both study outcomes. *Time spent working for COVida* was calibrated directly (Figure S65). In-set membership was when an activista spent 20 or more hours per month working for COVida (

Figure S76). Out-of-set membership was when an activista spent 5 or fewer hours per month working for COVida. The crossover point was when an activista spent 15 hours per month working for COVida.

**Figure S65.** Raw data for *time spent working for COVida* by activista

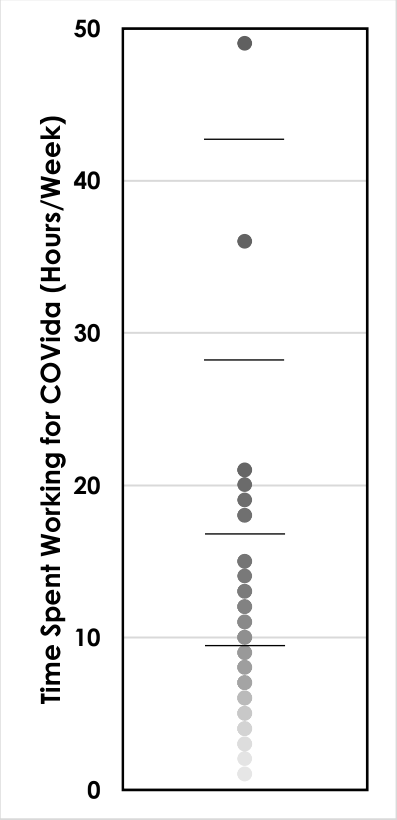

**Figure S76.** Direct calibration for *time spent working for COVida*

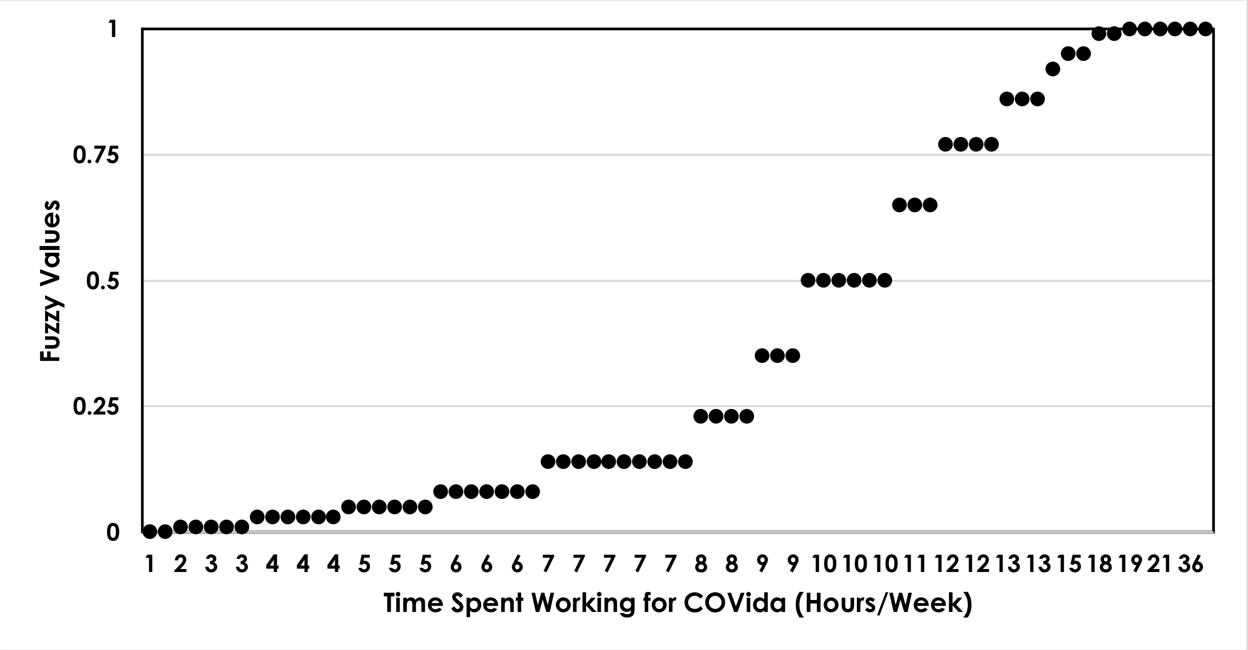


## Training

*Training* was defined as the number of training days provided to the activista, their chefe, and their chefe's supervisor. A greater amount of training was hypothesized to positively influence effective case management and, therefore, a positive change in both study outcomes. The overall *training* condition was aggregated from 3 micro-conditions: activista training, chefe training, and supervisor training. Since the activistas have the most direct influence on each case, it is likely that their training is more influential in the overall case management than that of the chefes and supervisors. Consequently, the training condition used a weighted sum from the 3 micro-conditions, where the fuzzy value for the activista training was weighted by 0.5, and the fuzzy values for the chef training and supervisor training were weighted by 0.25 each (Table S43). In-set membership was when training lasted for 12 or more days (Table S54, Table S65, Table S76). Out-of-set membership was when training lasted for 3 or fewer days.

**Table S43.** Aggregation method for *training*

| Weighting | Micro-condition |
|---|---|
| 0.5 | Activista training |
| 0.25 | Chefe training |
| 0.25 | Supervisor training |

**Table S54.** Indirect calibration for *activista training* (micro-condition of *training*)

| Fuzzy Value | Calibration |
|---|---|
| 1 | The activista's training lasted for 12 or more days. |
| 0.67 | The activista's training lasted from 8 to 11 days. |
| 0.33 | The activista's training lasted from 4 to 7 days. |
| 0 | The activista's training lasted for 3 days or less. |

**Table S65.** Indirect calibration for *chefe training* (micro-condition of *training*)

| Fuzzy Value | Calibration |
|---|---|
| 1 | The chef's training lasted for 12 or more days. |
| 0.67 | The chef's training lasted from 8 to 11 days. |
| 0.33 | The chef's training lasted from 4 to 7 days. |
| 0 | The chef's training lasted for 3 days or less. |

**Table S76.** Indirect calibration for *supervisor training* (micro-condition of *training*)

| Fuzzy Value | Calibration |
|---|---|
| 1 | The supervisor's training lasted for 12 or more days. |
| 0.67 | The supervisor's training lasted from 8 to 11 days. |
| 0.33 | The supervisor's training lasted from 4 to 7 days. |
| 0 | The supervisor's training lasted for 3 days or less. |

Work Experience

*Work experience* was defined as the total number of months an activista had been employed as an activista either with COVida or another organization. More work experience was hypothesized to positively influence effective case management and, therefore, a positive change in both study outcomes. In-set membership was when an activista had worked as an activista for 24 or more months (Table S87). Out-of-set membership was when an activista had worked as an activista for 6 months or less.

**Table S87.** Indirect calibration for *work experience*

| Fuzzy Value | Calibration |
|---|---|
| 1 | Activista has worked as an activista for 24 or more months. |
| 0.67 | Activista has worked as an activista for 12 to 23 months. |
| 0.33 | Activista has worked as an activista for 7 to 11 months. |
| 0 | Activista has worked as an activista for 6 months or less. |
